# Efficacy of acupuncture and pharmacological therapies for vascular cognitive impairment with no dementia: a network meta-analysis

**DOI:** 10.3389/fnagi.2023.1181160

**Published:** 2023-06-15

**Authors:** Ruiyu Li, Congcong Xu, Pengyu Zhong, Ke Wang, YinXiang Luo, Lingyong Xiao, Xiaoyu Dai, Jingxian Han, Xuezhu Zhang

**Affiliations:** ^1^Department of Acupuncture and Moxibustion, First Teaching Hospital of Tianjin University of Traditional Chinese Medicine, Tianjin, China; ^2^National Clinical Research Center for Chinese Medicine Acupuncture and Moxibustion, Tianjin, China; ^3^Department of Cardiology, Nanchong Central Hospital, Nanchong, China

**Keywords:** network meta-analysis, vascular cognitive impairment with no dementia, acupuncture, pharmacological therapy, efficacy

## Abstract

**Background and objective:**

Vascular cognitive impairment with no dementia (VCIND) is considered to be the prodromal stage of vascular dementia, characterized by insidious onset. Although acupuncture and drug therapies are effective, the optimal therapy for VCIND remains to be further determined. Therefore, we conducted a network meta-analysis to compare the effectiveness of acupuncture therapies and current common medicines for VCIND.

**Methods:**

We searched eight electronic databases to identify eligible randomized controlled trials of patients with VCIND treated by acupuncture or drug therapies. The primary outcome was Montreal Cognitive Assessment, and the secondary outcome was Mini-Mental State Examination. We conducted the network meta-analysis within a Bayesian framework. Weighted mean difference with 95% confidence intervals were applied as effect sizes to continuous data for all outcomes. Sensitivity analysis was done to assess the robustness of the findings, and we also carried out a subgroup analysis based on age. We assessed the risk of bias using the Risk of Bias 2.0 tool and applied the Grade of Recommendation Assessment, Development and Evaluation (GRADE) to assess the quality of the outcomes. This study was registered with PROSPERO, number CRD42022331718.

**Results:**

A total of 33 studies with 14 interventions were included, including 2603 participants. In terms of the primary outcome, manual acupuncture plus herbal decoction was considered to be the most effective intervention (*P* = 91.41%), followed by electroacupuncture (*P* = 60.77%) and manual acupuncture plus piracetam (*P* = 42.58%), whereas donepezil hydrochloride ranked the least efficacious intervention (*P* = 54.19%). For the secondary outcome, electroacupuncture plus nimodipine was considered to be the most effective intervention (*P* = 42.70%), followed by manual acupuncture plus nimodipine (*P* = 30.62%) and manual acupuncture (*P* = 28.89%), whereas nimodipine ranked the least efficacious intervention (*P* = 44.56%).

**Conclusion:**

Manual acupuncture plus herbal decoction might be the most effective intervention for VCIND. The combination of acupuncture and drug therapy had a tendency to perform better than monotherapy in terms of clinical outcomes.

**Systematic review registration:**

https://www.crd.york.ac.uk/PROSPERO/display_record.php?RecordID=331718, identifier: CRD42022331718.

## Introduction

Vascular cognitive impairment with no dementia (VCIND) is a common type of vascular cognitive impairment (VCI), characterized by mild impairment of attention and executive function. It is generated by underlying vascular causes and considered as a prodromal stage of vascular dementia (VD). The progress of VCIND can be insidious and slow, often accompanied by cognitive impairments in multiple brain regions. In China, 20.8% of individuals over 65 years suffer from mild cognitive impairment (MCI), a concept similar to the narrow sense of CIND, and the vascular-related subtype accounts for 42.0% of the total cases, namely VCIND is the most common subtype (Jia et al., 2014). Patients with VCIND are at high risk of developing dementia with ~50% of them progressing to dementia after 5 years (Wentzel et al., 2001). Fortunately, available evidence suggests that the progress of VCIND can be delayed or even reversed by early detection and intervention (Gorelick et al., 2011).

However, evidence-based therapies for patients with VCIND are lacking (Langa and Levine, 2014). Common medications for cognitive impairment bare several limitations. Cholinesterase inhibitor such as donepezil hydrochloride has a propensity to increase gastrointestinal adverse effects, the neuroprotective agent piracetam can cause anxiety, insomnia or agitation, and the clinical efficacy of the calcium antagonist nimodipine is still uncertain (Wang et al., 2016; Cohen et al., 2020). As a characteristic traditional Chinese medicine (TCM) therapy, herbal decoction demonstrates a great potential for curing VCIND patients, but the treatment based on syndrome differentiation has increased the difficulty of its promotion. Acupuncture, a therapy unique to TCM, features minimally invasive techniques. Nowadays, acupuncture therapy has derived a variety of acupuncture techniques, including manual acupuncture, electroacupuncture, warm acupuncture and so on. These acupuncture therapies have shown promising clinical efficacy in combination with drug therapies or even used alone. Given such a wide variety of therapy options, it may be difficult for physicians and patients to make an informed clinical decision. Although a previous meta-analysis has cautiously shown that acupuncture therapy is more effective in treating VCIND when compared to conventional or pharmacological therapies (Deng and Wang, 2016), the optimal intervention for VCIND still remains to be determined.

In view of the urgent need for the optimal VCIND therapy, this study was committed to comparing the effectiveness of acupuncture therapies and current common medicines for VCIND, so as to provide the evidence for selection of rational therapeutic regimen in the clinic.

## Methods

### Search strategy

We carried out this Bayesian network meta-analysis following the Preferred Reporting Items for Systematic Reviews and Meta-Analysis (PRISMA) statement principles and the PRISMA extension statement for network meta-analysis (Hutton et al., 2015; Page et al., 2021). We searched PubMed, Excerpta Medical Database, Web of Science, the Cochrane Library, Chinese Biomedical Literature Database, China National Knowledge Infrastructure, China Science and Technology Journal Database and Wan fang Database from the date of their inception to March 12, 2022. Ongoing trials were searched in the United States clinical trials registry, WHO International Trials Registry Platform on March 12th, 2022. Languages were limited to English and Chinese. The major search terms of PubMed were as follows: “vascular cognitive impairment with no dementia” or “VCIND” and “acupuncture” or “acupuncture therapy” or “acupuncture points” and “randomized controlled trials.” The details of the search strategy are summarized in Supplementary Tables S1–S4. In addition, relevant systematic reviews identified by the strategy were checked in case there was any missing literature by hand screening the reference lists. All the references were managed by Endnote. This study is registered with PROSPERO, CRD42022331718.

### Inclusion and exclusion criteria

The inclusion criteria of the study met the following requirements: (a) randomized controlled trials (RCTs); (b) patients were diagnosed with VCI regardless of their age, sex, ethnicity or education but did not meet the criteria for dementia; (c) interventions for the experimental group were acupuncture therapies or acupuncture therapies in combination with pharmacotherapy, and drugs were limited to nimodipine, donepezil hydrochloride, piracetam and herbal decoction; (d) control group was treated with nimodipine, donepezil hydrochloride, piracetam or herbal decoction; and (e) the primary outcome was Montreal Cognitive Assessment (MoCA), and the secondary outcome was Mini-Mental State Examination (MMSE). The exclusion criteria included: (a) literature reviews, comments, case reports, cohort studies, cross-sectional studies, case-control studies or animal experiments; (b) duplicate studies; (c) studies with improper randomization methods; and (d) studies without full-texts or sufficient information.

### Selection criteria and data extraction

We used the software EndNote X9 to eliminate duplicate studies after the search was completed. Titles and abstracts were screened by two researchers (RYL, CCX) based on inclusion and exclusion criteria. The full-text articles of shortlisted studies were then assessed for eligibility. Detailed data were extracted from the final eligible studies by two researchers (RYL, KW) independently. Then the two researchers (RYL, KW) recorded study ID (author, publication year), basic information of participants (diagnose criteria, mean age, gender, nationality, disease duration), sample size, intervention, course of treatment, outcome, duration of follow-up period and number of shedding people. Any divergences in the process were resolved by discussion or consultation with the third researcher (YXL).

### Risk of Bias assessment and GRADE quality of evidence

Two independent researchers (RYL, CCX) used the Cochrane risk of bias (RoB 2) tool to assess the risk of bias in the included RCTs based on five areas: randomization process, deviations from intended interventions, missing outcome data, measurement of the outcome and selection of the reported result. The risk of bias was defined as low risk, some concerns and high risk. Disagreements were submitted to and judged by the third researcher (KW). Besides, we applied Grades of Recommendations Assessment, Development and Evaluation (GRADE) to evaluate the quality of outcomes according to direct, indirect and network comparison, respectively (Puhan et al., 2014; Salanti et al., 2014).

### Statistical analysis

We used R 4.1.3, JAGS 4.3.0 and STATA 16.0 to conduct statistical analysis, in which the packages “gemtc,” “rjags,” “robvis,” “ggplot2” and “network graphs” were invoked. Weighted mean difference with 95% confidence intervals were applied as effect sizes to continuous data for all outcomes. *P*-value < 0.05 was regarded as statistically significant. The selection of random-effects or fixed-effects model was based on deviance information criteria (DIC). When the difference between the DIC values of the random-effects model and the fixed-effects model was >10, we selected the model with the lower DIC value. We used the Markov chain Monte Carlo algorithm for Bayesian inference. For each model, four chains were run for 20,000 warm-up iterations and then 50,000 sampling iterations. The convergence of the models was ensured with Brooks–Gelman–Rubin diagnostic, trace and density plots. Convergence was achieved when the potential scale reduction factor was close to 1.0. We carried out the evaluation of inconsistency by using a node-splitting analysis, in which a *P*-value < 0.05 indicated significant inconsistency. Heterogeneity was examined using the standard *I*^2^ test (with *I*^2^ > 50% indicating substantial heterogeneity). Sensitivity analysis was done to assess the robustness of the findings, and we also carried out a subgroup analysis based on age.

## Results

### Study selection and study characteristics

**Figure 1:**
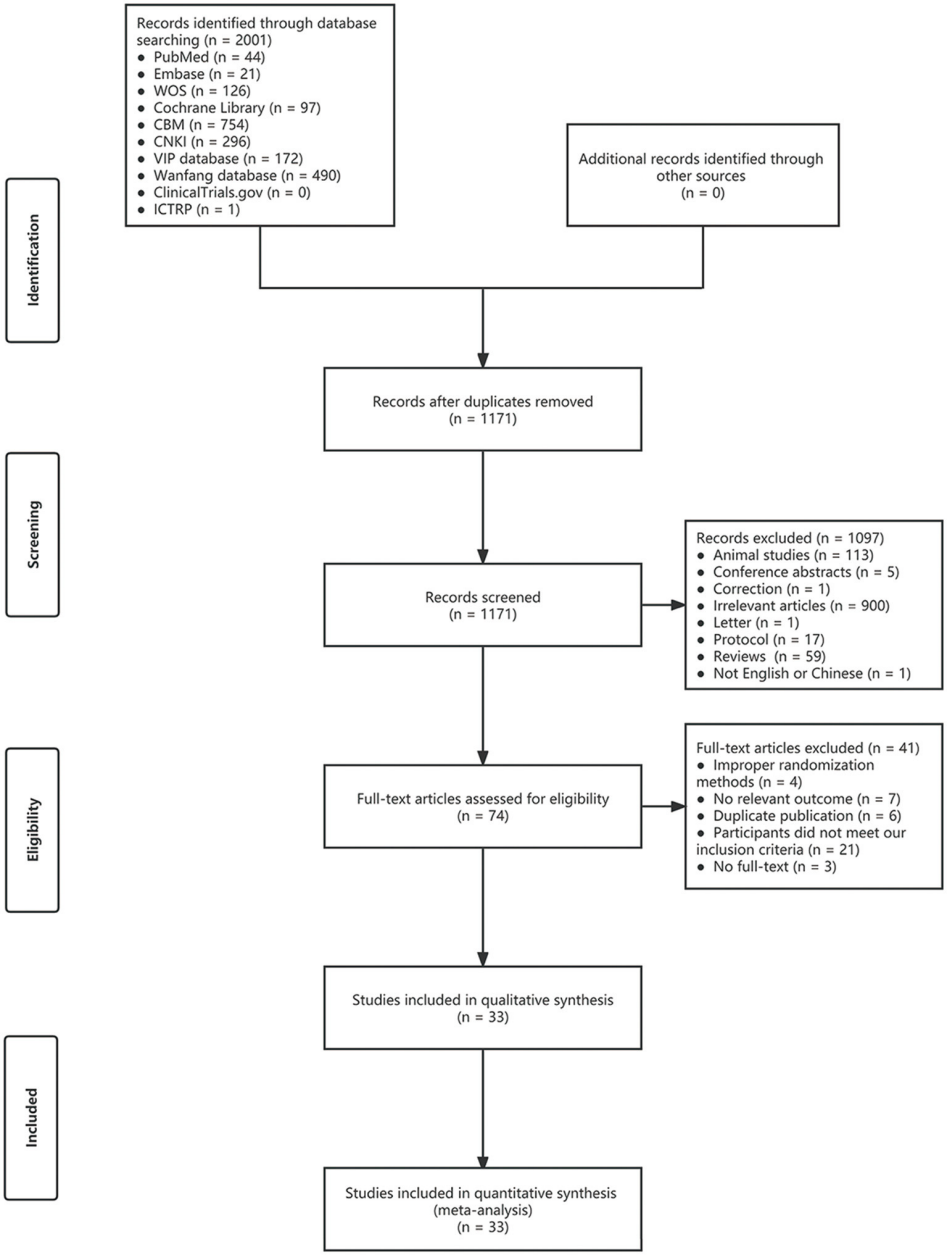
Literature search and selection.

The process of literature screening and study selection is shown in Figure 1. A total of 2,001 articles were searched from 8 databases. Finally, 33 randomized controlled trials met the inclusion and exclusion criteria out of 74 full-text articles reviewed (Yu and Han, 2007; Jiao, 2011; Kong, 2011; Li et al., 2012, 2018; Shao, 2012; Feng et al., 2013; Chen, 2014; Wang et al., 2014, 2016, 2017; Zhang, 2014, 2018, 2021; Zheng, 2014; Dong, 2015; Yang et al., 2015; Sun et al., 2016; Wang, 2016; Liu and Xie, 2017; Sun, 2017; Yu, 2017; Zhao et al., 2017; Wang and Li, 2018; Zhang and Li, 2018; Luo et al., 2019; Xu and Zhang, 2019; Zhang et al., 2019; Meng et al., 2020; Ni et al., 2020; Yu et al., 2020; Bai et al., 2021; Liu et al., 2021).

The characteristics of the included studies and patients are presented in Table 1 and Supplementary Table S5, and the characteristics of treatments are shown in Table 2. A total of 2,603 participants participated in these studies, which were published between 2007 and 2021. Of these 33 RCTs, 4 were three-arm trials and 29 were two-arm trials. A total of 14 interventions were applied, including manual acupuncture, electroacupuncture, nimodipine, donepezil hydrochloride, piracetam, herbal decoction, manual acupuncture plus electroacupuncture, manual acupuncture plus nimodipine, manual acupuncture plus donepezil hydrochloride, manual acupuncture plus piracetam, manual acupuncture plus herbal decoction, electroacupuncture plus nimodipine, warm acupuncture plus nimodipine and manual acupuncture plus electroacupuncture plus nimodipine. These studies all were from China and had clear diagnostic and outcome criteria.

**Table 1:** Characteristics of included studies.

**REferences**	**Sample size (E/C)**	**Treatment (E/C)**	**Course of treatment (months)**	**Outcome**	**Follow-up (months)**	**Number of shedding people (%)**	**Diagnostic criteria**
Yu and Han (2007)	34/33	A/C	3	②	–	3 (4.48%)	Expert recommendation
Jiao (2011)	30/30	I/D	2	②	–	0 (0.00%)	Expert recommendation
Kong (2011)	30/30	I/D	1.5	②	–	0 (0.00%)	Expert recommendation
Li et al. (2012)	50/50	N/C	3	②	–	6 (6%)	CPT-2006
Shao (2012)	30/30	G/C	1.5	①②	–	0 (0.00%)	CPT-2006
Feng et al. (2013)	24/24	I/D	1.5	②②	–	0 (0.00%)	EC-2007
Chen (2014)	20/20	G/C	1	②②	–	0 (0.00%)	CPT-2006
Wang et al. (2014)	30/30	H/C	3	①	–	0 (0.00%)	Expert recommendation
Zhang (2014)	30/30	N/C	1	②②	–	0 (0.00%)	Expert recommendation
Zheng (2014)	30/30	H/C	2	②②	–	0 (0.00%)	Expert recommendation
Dong (2015)	40/40	G/C	1	②②	–	0 (0.00%)	CPT-2006
Yang et al. (2015)	36/36	H/C	3	①	–	0 (0.00%)	GDT-2011
Sun et al. (2016)	40/40	I/D	3	①	–	0 (0.00%)	CPT-2006
Wang (2016)	32/32	A/C	2	①	–	3 (4.69%)	GDT-2011
Wang et al. (2016)	42/42/42	A/H/C	3	①	3	7 (5.56%)	GDT-2011
Liu and Xie (2017)	60/30	A/C	1	①②	–	0 (0.00%)	CPT-2006
Sun (2017)	33/33	L/C	1	①②	–	6 (9.09%)	GDT-2011
Wang et al. (2017)	20/20	A/C	2	①②	–	0 (0.00%)	VCIHS-2006
Yu (2017)	30/30	I/D	3	①	3	5 (8.33%)	VCIHS-2006
Zhao et al. (2017)	17/17	A/C	3	①②	–	0 (0.00%)	GDT-2011
Li et al. (2018)	30/30	H/C	1	①	–	0 (0.00%)	GDT-2011
Zhang (2018)	30/30	M/C	1	①	–	0 (0.00%)	ECM-2017
Zhang and Li (2018)	40/40	B/C	2	①②	–	0 (0.00%)	MCIMP-2006
Wang and Li (2018)	64/64	H/C	2.5	①②	–	0 (0.00%)	MCIDI-2004
Luo et al. (2019)	100/100	G/C	2	①②	3	12 (6.00%)	Expert recommendation
Xu and Zhang (2019)	56/38	A/D	2	①	–	16 (17.02%)	DSM-V
Zhang et al. (2019)	42/41/39	A/K/F	2	①	–	17 (13.93%)	DSM-V
Meng et al. (2020)	61/61	I/D	1	①②	–	0 (0.00%)	Expert recommendation
Ni et al. (2020)	31/30/31	A/J/E	2	①	–	2 (2.17%)	EC-2007
Yu et al. (2020)	40/40	I/D	3	①	–	0 (0.00%)	Expert recommendation
Bai et al. (2021)	33/33	I/D	2	①②	–	5 (7.58%)	CGD-2016
Liu et al. (2021)	34/34/34	A/B/E	1	①	–	0 (0.00%)	CGD-2016
Zhang (2021)	35/35	A/D	1.5	①②	–	2 (2.86%)	DSM-V

E, experimental group; C, control group.

Treatment: A, manual acupuncture; B, electroacupuncture; C, nimodipine; D, donepezil hydrochloride; E, piracetam; F, herbal decoction; G, manual acupuncture plus electroacupuncture; H, manual acupuncture plus nimodipine; I, manual acupuncture plus donepezil hydrochloride; J, manual acupuncture plus piracetam; K, manual acupuncture plus herbal decoction; L, electroacupuncture plus nimodipine; M, warm acupuncture plus nimodipine; N, manual acupuncture plus electroacupuncture plus nimodipine.

Outcome: ①, Montreal Cognitive Assessment, MoCA; ②, Mini-Mental State Examination, MMSE.

Diagnostic criteria: CPT-2006, 2006 China Expert Consensus on Prevention and Treatment of Cognitive Dysfunction; EC-2007, 2007 Expert Consensus on Vascular Cognitive Impairment; GDT-2011, 2011 Guidance on Diagnosis and Treatment of Vascular Cognitive Impairment; VCIHS-2006, 2006 National Institute of Neurological Disorders and Stroke-Canadian stroke Network Vascular cognitive Impairment Harmonization standards; ECM-2017, 2017 Expert Consensus on The Management of Cognitive Disorders After Stroke; MCIMP-2006, 2006 Mild cognitive impairment (MCI)in medical practice; MCIDI-2004, 2004 Mild cognitive impairment diagnosis and intervention; DSM-V, Diagnostic and Statistical Manual of Mental Disorders-5th; CGD-2016, 2016 Chinese Guidelines for the Diagnosis of Vascular Mild Cognitive Impairment.

**Table 2:** Characteristics of interventions of included studies.

**References**	**Design**	**Treatment**	
		**E**	**C**
Yu and Han (2007)	Two-arm	Manual acupuncture (RN17, RN12, RN6, SP10, ST36, SJ5, five times a week)	Nimodipine 30 mg, three times a day
Jiao (2011)	Two-arm	Manual acupuncture (DU20, EX-HN5, EX-HN1, once a day, 40 min once) plus donepezil hydrochloride (5 mg, once a day)	Donepezil hydrochloride 5 mg, once a day
Kong (2011)	Two-arm	Manual acupuncture (DU20, EX-HN1, DU24, GB20, LR3, HT7, KI3, BL58, SP3, ST40, once a day, 30 min once) plus donepezil hydrochloride (5 mg, once a day)	Donepezil hydrochloride 5 mg, once a day
Li et al. (2012)	Two-arm	Manual acupuncture plus electroacupuncture (DU20, DU24, BL4, EX-HN1, GB20, PC6, LI4, ST36, SP6, KI3, KI6, five times a week, 30 min once) plus nimodipine (30 mg, three times a day)	Nimodipine 30 mg, three times a day
Shao (2012)	Two-arm	Manual acupuncture plus electroacupuncture (DU20, DU24, KI3, HT7, five times a week, 30 min once)	Nimodipine 30 mg, three times a day
Feng et al. (2013)	Two-arm	Manual acupuncture (EX-HN1, DU20, DU23, DU24, EX-HN3, GB20, KI3, GB39, HT7, ST40, BL50, SP3, LR3, LI4, once a day, 40 min once) plus donepezil hydrochloride (5 mg, once a day)	Donepezil hydrochloride 5 mg, once a day
Chen (2014)	Two-arm	Manual acupuncture plus electroacupuncture (EX-HN1, DU24, GB13, once a day, 30 min once)	Nimodipine 30 mg, three times a day
Wang et al. (2014)	Two-arm	Manual acupuncture (DU20, DU24, EX-HN1, LI4, LR3, SP6, ST40, RN12, ST36, once a day, 30 min once) plus nimodipine (30 mg, three times a day)	Nimodipine 30 mg, three times a day
Zhang (2014)	Two-arm	Manual acupuncture plus electroacupuncture (DU20, EX-HN1, DU24, GB20, SP6, RN4, KI3, HT7, PC6, five times a week, 30 min once) plus nimodipine (20 mg, three times a day)	Nimodipine 20 mg, three times a day
Zheng (2014)	Two-arm	Manual acupuncture (DU20, DU24, DU14, DU11, DU4, BL23, KI3, once a day, 30 min once) plus nimodipine (30 mg, three times a day)	Nimodipine 30 mg, three times a day
Dong (2015)	Two-arm	Manual acupuncture plus electroacupuncture (EX-HN1, DU24, GB13, once a day, 30 min once)	Nimodipine 30 mg, three times a day
Yang et al. (2015)	Two-arm	Manual acupuncture (DU20, EX-HN1, ST2, GB20, GB12, BL10, HT1, PC6, DU26, SP6, LR3, ST40, once a day) plus nimodipine (30 mg, three times a day)	Nimodipine 30 mg, three times a day
Sun et al. (2016)	Two-arm	Manual acupuncture (HT7, KI3, BL58, SP3, ST40, LR3, once a day, 40 min once) plus donepezil hydrochloride (2.5 mg, once a day)	Donepezil hydrochloride, 2.5 mg, once a day
Wang (2016)	Two-arm	Manual acupuncture (DU22, DU24, DU20, EX-HN1, GB4, GB6, GB7, GB8, six times a week, 40 min once)	Nimodipine 20 mg, three times a day
Wang et al. (2016)	Three-arm	Manual acupuncture (DU20, EX-HN1, ST2, GB20, GB12, BL10, DU26, HT7, PC6, ST40, SP6, LR3, six times a week, 30 min once) plus nimodipine (30 mg, three times a day); Manual acupuncture (DU20, EX-HN1, ST2, GB20, GB12, BL10, DU26, HT7, PC6, ST40, SP6, LR3, six times a week, 30 min once)	Nimodipine 30 mg, three times a day
Liu and Xie (2017)	Two-arm	Manual acupuncture (DU24, DU20, DU18, DU17, DU16, DU14, DU11, once a day, 40 min once; EX-HN3, EXHN1, DU20, GB20, KI3, GB39, LI4, LR3, once a day, 40 min once)	Nimodipine 30 mg, three times a day
Sun (2017)	Two-arm	Electroacupuncture (DU20, DU14, DU16, ST36, ST40, LI4, LR3, once a day, 30 min once) plus nimodipine (30 mg, three times a day)	Nimodipine 30 mg, three times a day
Wang et al. (2017)	Two-arm	Manual acupuncture (DU3, DU4, DU9, DU11, DU12, DU14, DU15, DU16, DU20, EX-B2, GB20, six times a week, 30 min once)	Nimodipine 30 mg, three times a day
Yu (2017)	Two-arm	Manual acupuncture (DU20, DU24, HT7, HT5, KI3, GB39, ST36, twice a week, 30 min once) plus donepezil hydrochloride (5 mg, once a day)	Donepezil hydrochloride 5 mg, once a day
Zhao et al. (2017)	Two-arm	Manual acupuncture (DU20, EX-HN1, GB20, RN12, SP10, ST36, six times a week, 30 min once)	Nimodipine 30 mg, three times a day
Li et al. (2018)	Two-arm	Manual acupuncture (DU24, DU16, DU20, ST36, DU14, ST40, LR3, LI4, once a day, 30 min once) plus nimodipine (30 mg, three times a day)	Nimodipine 30 mg, three times a day
Zhang (2018)	Two-arm	Warm acupuncture (BL23, RN4, DU16, JT7, KI3, LI10, ST36, DU26, DU24, GB18, ST40, once a day, 30 min once) plus nimodipine (40 mg, three times a day)	Nimodipine 40 mg, three times a day
Zhang and Li (2018)	Two-arm	Electroacupuncture (DU20, Bagua points, five times a week, 30 min once)	Nimodipine 30 mg, three times a day
Wang and Li (2018)	Two-arm	Manual acupuncture (DU26, PC6, SP6, DU20, DU21, DU22, DU24, GB13) plus nimodipine (40 mg, three times a day)	Nimodipine 40 mg, three times a day
Luo et al. (2019)	Two-arm	Manual acupuncture plus electroacupuncture (GB20, DU16, DU14, Xiangsihua poionts (Extra), five times a week, 30 min once)	Nimodipine 30 mg, three times a day
Xu and Zhang (2019)	Two-arm	Manual acupuncture (DU26, PC6, SP6, EX-HN1, GB39, KI3, twice a day)	Donepezil hydrochloride 5 mg, once a day
Zhang et al. (2019)	Three-arm	Manual acupuncture (DU26, PC6, SP6, EX-HN1, GB39, KI3, twice a day) plus herbal decoction (Supplemented Guipi-tang 150 ml, twice a day); Manual acupuncture (DU26, PC6, SP6, EX-HN1, GB39, KI3, twice a day)	Herbal decoction (Supplemented Guipi-tang 150 ml, twice a day)
Meng et al. (2020)	Two- arm	Manual acupuncture (DU20, DU24, DU26, EX-HN3, DU16, KI3, LR3, ST40, PC6, SP6, once a day, 30 min once) plus donepezil hydrochloride (5 mg, once a day)	Donepezil hydrochloride 5 mg, once a day
Ni et al. (2020)	Three-arm	Manual acupuncture (acupoints selected by Zi Wu Liu Zhu Na Jia method, once a day, 30 min once) plus piracetam (0.4 g, three times a day); Manual acupuncture (acupoints selected by Zi Wu Liu Zhu Na Jia method, once a day, 30 min once)	Piracetam 0.4 g, three times a day
Yu et al. (2020)	Two-arm	Manual acupuncture (HT7, DU24, DU20, KI3, HT5, GB39, ST36, twice a week, 30 min once) plus donepezil hydrochloride (5 mg, once a day)	Donepezil hydrochloride 5 mg, once a day
Bai et al. (2021)	Two-arm	Manual acupuncture (DU20, DU24, DU16, DU14, DU9, DU4, DU3, once every 2 days, 40 min once) plus donepezil hydrochloride (10 mg, once a day)	Donepezil hydrochloride 10 mg, once a day
Liu et al. (2021)	Three-arm	Electroacupuncture (DU20, EX-HN1, ST8, GB20, BL23, BL35, Gongxue points, once a day, 30 min once); Manual acupuncture (DU20, EX-HN1, ST8, GB20, BL23, BL35, Gongxue points, once a day, 30 min once)	Piracetam 0.8 g, three times a day
Zhang (2021)	Two-arm	Manual acupuncture (DU24, DU20, PC6, HT5, SP6, LR3, six times a week, 30 min once)	Donepezil hydrochloride 5 mg, once a day

E, experimental group; C, control group.

Supplemented Guipi-tang: Atractylodis Macrocephalae Rhizoma (Bai Zhu) 9.0 g, Ginseng Radix (Ren Shen) 12.0 g, Astragali Radix (Huang Qi) 12.0 g, Angelicae Sinensis Radix (Dang Gui) 9.0 g, Poria cocos (Fu Ling) 9.0 g, Polygalae Radix (Yuan Zhi) 9.0 g, Zizyphi Spinosi Semen (Suan Zao Ren) 9.0 g, Aucklandiae Radix (Mu Xiang) 9.0 g, Longan Arillus (Long Yan Rou) 9.0 g, Zingiberis Rhizoma Recens (Sheng Jiang) 6.0 g, Jujubae Fructus (Da Zao) 6.0 g, Glycyrrhizae Radix (Gan Cao) 6.0 g, Ligustici Rhizoma (Chuan Xiong) 15.0 g, Carthami Flos (Hong Hua) 12.0 g, Lumbricus (Di Long) 6.0 g.

### Risk of bias for research quality evaluation

Among the 33 RCTs, 28 RCTs had some concerns with randomization process, and only 5 RCTs mentioned proper allocation concealment. In terms of deviations from intended interventions, 31 RCTs had some concerns, while 2 RCTs had a high risk of bias considering their high dropout rates. As for missing outcome data, 31 trials had a low risk of bias, and only 2 trials had a high risk of bias due to lack of sufficient data. All included RCTs had a low risk of bias for measurement of the outcome. Besides, all RCTs included had some concerns in selection of the reported result because of insufficient information on pre-specified analysis protocols. The risk of bias assessment is summarized in Supplementary Figure S1.

### Network evidence plot

**Figure 2:**
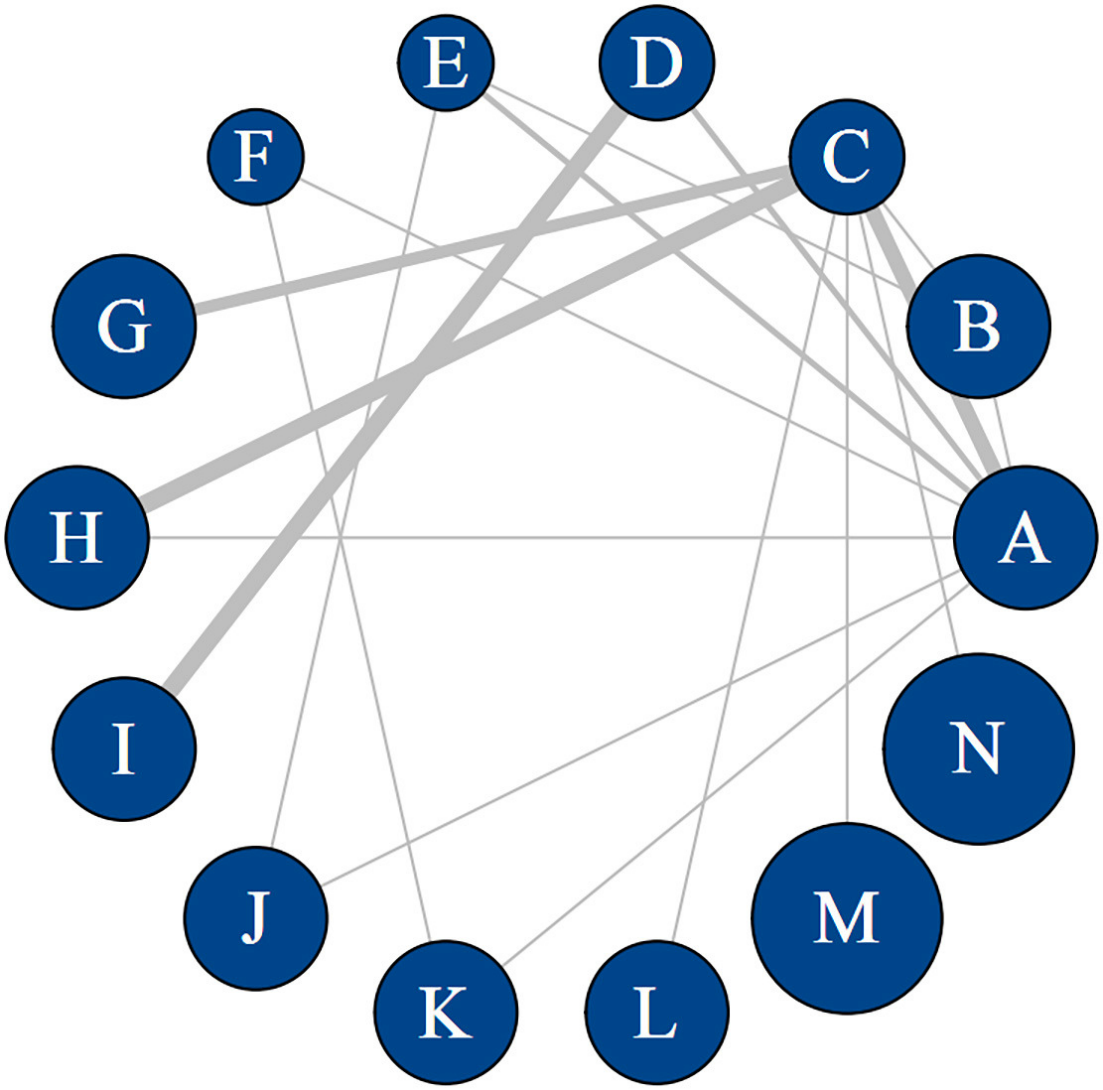
Network map of different interventions for MoCA. Width of the lines is proportional to the number of trials comparing every pair of treatments. Size of every circle is proportional to the number of randomly assigned participants (i.e., sample size). A: manual acupuncture; B: electroacupuncture; C: nimodipine; D: donepezil hydrochloride; E: piracetam; F: herbal decoction; G: manual acupuncture plus electroacupuncture; H: manual acupuncture plus nimodipine; I: manual acupuncture plus donepezil hydrochloride; J: manual acupuncture plus piracetam; K: manual acupuncture plus herbal decoction; L: electroacupuncture plus nimodipine; M: warm acupuncture plus nimodipine; N: manual acupuncture plus electroacupuncture plus nimodipine.

The network evidence plots (Figure 2 and Supplementary Figure S2) showed the networks of comparisons for the primary outcome, MoCA, and the secondary outcome, MMSE. MoCA was reported in 29 studies including 2,316 patients and 14 interventions. MMSE was reported in 20 studies including 1,531 patients and 9 interventions. The width of the lines indicates the number of studies comparing the connected interventions, and the size of nodes indicates the number of samples involved in relevant interventions.

### Evaluation of statistical model

The consistency model was fitted to conduct network meta-analysis, and random model was selected because the DIC value of the random model was less than that of the fixed model. In terms of the convergence of models, convergence diagnostic plots (Supplementary Figures S3, S4) showed that both median value of the reduction factor and 97.5% tended to be stable after 40,000 iterations, and the parameter potential scale reduction factor moved close to 1, indicating a satisfactory convergence of the models. Besides, density plots and trace plots (Supplementary Figures S5, S6) also indicated a satisfactory convergence.

### Primary and secondary outcomes

**Figure 3:**
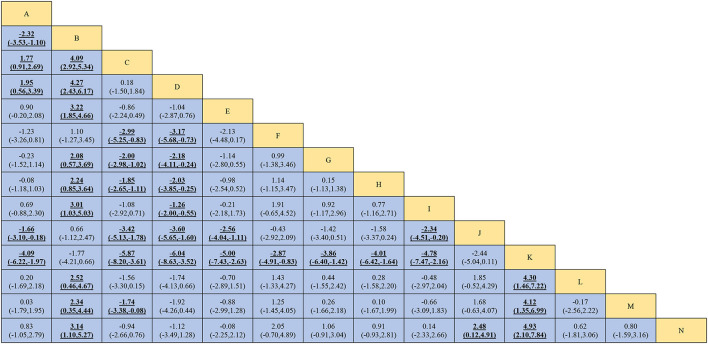
Network meta-analysis Comparisons for MoCA. Interventions are reported in alphabetical order. Data are MDs (95% CI) of the column-defining intervention compared to the row-defining intervention. For MoCA, 95% CI doesn't contain 0 and MDs higher than 0 favor the column-defining treatment (i.e., the first in alphabetical order). Significant results are in bold and underscored. Manual acupuncture plus herbal decoction (MDs ranging between 2.87 and 6.04), electroacupuncture (MDs ranging between 2.08 and 4.27) and manual acupuncture plus piracetam (MDs ranging between 1.66 and 3.60) are more effective than other interventions. Nimodipine (MDs ranging between −1.74 and −5.87) and donepezil hydrochloride (MDs ranging between −1.26 and −6.04) are among the least efficacious interventions. MD, mean difference; CI, confidence interval. Intervention: A: manual acupuncture; B: electroacupuncture; C: nimodipine; D: donepezil hydrochloride; E: piracetam; F: herbal decoction; G: manual acupuncture plus electroacupuncture; H: manual acupuncture plus nimodipine; I: manual acupuncture plus donepezil hydrochloride; J: manual acupuncture plus piracetam; K: manual acupuncture plus herbal decoction; L: electroacupuncture plus nimodipine; M: warm acupuncture plus nimodipine; N: manual acupuncture plus electroacupuncture plus nimodipine.

We synthesized all studies to assess the differences in interventions. The results for MoCA and MMSE were shown in Figure 3 and Supplementary Figure S7, respectively. Data are MDs (95% CI) of the column-defining intervention compared to the row-defining intervention. For MoCA and MMSE, 95% CI doesn't contain 0 and MDs higher than 0 favor the column-defining treatment. Significant results are in bold and underscored. For MoCA, manual acupuncture plus herbal decoction, electroacupuncture and manual acupuncture plus piracetam were more effective than other interventions (MDs ranging between 1.66 and 6.04), whereas nimodipine and donepezil hydrochloride were among the least efficacious interventions (MDs ranging between −1.26 and −6.04). For MMSE, nimodipine and donepezil hydrochloride were among the least efficacious interventions (MDs ranging between −1.39 and −2.40). To observe the robustness of the results, we also did a sensitivity analysis. After removing studies that did not mention explicit randomization methods and blinding information, the results did not change substantially.

### Ranking of interventions

**Figure 4:**
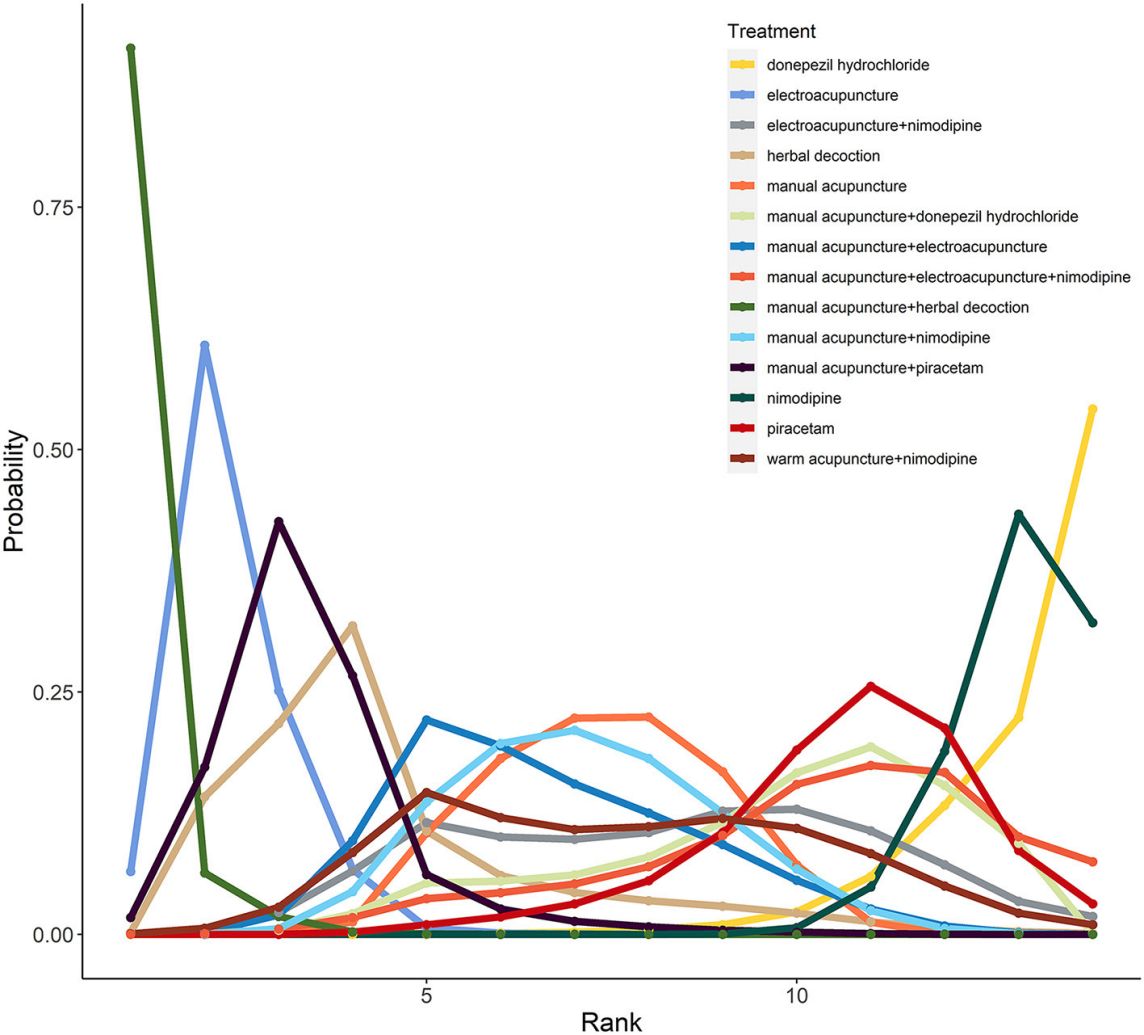
The rank probability of MoCA for included interventions. Manual acupuncture plus herbal decoction is the optimal strategy for improving MoCA scores (Probability of rank, 91%). Electroacupuncture and manual acupuncture plus piracetam rank second to third, respectively. Donepezil hydrochloride is the worst strategy in improving MoCA scores (Probability of rank, 54%).

The ranking of interventions for probability evaluation based on the Markov chain Monte Carlo theory is shown in Figure 4 and Supplementary Figure S8. For MoCA, of the 14 interventions for VCIND included in this study, manual acupuncture plus herbal decoction was considered to be the most effective intervention (*P* = 91.41%), followed by electroacupuncture (*P* = 60.77%) and manual acupuncture plus piracetam (*P* = 42.58%), whereas donepezil hydrochloride ranked the least efficacious intervention (*P* = 54.19%). For MMSE, of the 9 interventions for VCIND included in this study, electroacupuncture plus nimodipine was considered to be the most effective intervention (*P* = 42.70%), followed by manual acupuncture plus nimodipine (*P* = 30.62%) and manual acupuncture (*P* = 28.89%), whereas nimodipine ranked the least efficacious intervention (*P* = 44.56%). In addition, sensitivity analysis suggested that the results did not change substantially except that the least efficacious intervention for MMSE was changed from nimodipine to donepezil hydrochloride, so the relevant results should be interpreted with caution.

### Heterogeneity and inconsistency test

For heterogeneity test, the pooled results were shown in Supplementary Table S6. After removing studies that did not mention explicit randomization methods and blinding information, the heterogeneity among studies decreased significantly. The network *I*^2^ of A vs. B and A vs. C decreased from 54.03 to 24.77 and 96.84 to 0.00, respectively, suggesting that the quality of studies may be one of the factors affecting the heterogeneity between studies. In addition, subgroup analysis of people younger than 65 years suggested that age may also be a source of heterogeneity, as the heterogeneity between studies decreased significantly after the removal of studies with an average age above 65 years, with network *I*^2^ of A vs. C, A vs. H, and C vs. H decreasing from 96.84 to 0.00, 32.26 to 0.94, and 70.89 to 0.00, respectively. Besides, as shown in Supplementary Table S6, the results of the pairwise meta-analysis are basically the same as those of the network meta-analysis.

As for inconsistency test, the results of node-splitting model showed that for MoCA, there were inconsistencies in the comparisons of closed circles, and for MMSE, there were no comparisons to assess for inconsistency due to the absence of closed loops. Besides, for MoCA, the majority had a moderate evidence quality in terms of the direct evidence comparison and indirect evidence comparison, and all of the network comparison showed a moderate evidence quality. See Supplementary Table S7.

### Publication bias

The funnel plots of MoCA and MMSE were shown in Supplementary Figures S9, S10. Most of the scattered points were symmetrically distributed on both sides of the red indicator line, indicating no significant publication bias.

## Discussion

This network meta-analysis comparing 14 interventions for VCIND had the following findings. Manual acupuncture plus herbal decoction will likely be the most effective intervention for VCIND, leading to superior efficacy in terms of clinical outcome. Manual acupuncture plus herbal decoction, electroacupuncture and manual acupuncture plus piracetam might be relatively ideal options for VCIND. Monotherapy with nimodipine or donepezil hydrochloride is considered to be the least efficacious intervention for VCIND. In addition, the combination therapy of acupuncture therapies and pharmacotherapy might exert a larger efficacy than monotherapy. However, because the overall quality of evidence based on GRADE criteria was moderate, further well-designed randomized trials are warranted.

VCIND is of concern to clinicians because of its high incidence, insidious onset and high treatment costs. For now, the main issue for VCIND is to find the optimal therapy. Drugs that target cognitive function are among the most commonly prescribed therapies for VCIND. At present, nimodipine, donepezil hydrochloride and piracetam are commonly employed to treat VCIND. The binding sites of nimodipine are densely distributed in specific regions of the hippocampus, caudate nucleus and cerebral cortex, which may account for its role in learning and memory processes. Donepezil hydrochloride is a non-competitive, high-affinity, reversible acetylcholinesterase inhibitor, which improves cognition by increasing cholinergic activity. As for piracetam, its beneficial effects are usually associated with impaired brain function under conditions such as free radical damage and aging. However, these drugs often possess various significant adverse effects. Nimodipine may be associated with a risk of long-term hypotension, rash, diarrhea and bradycardia. Donepezil hydrochloride can cause gastrointestinal side effects, such as nausea and vomiting. Piracetam at pharmaceutical dosages may also generate anxiety, insomnia, agitation, depression, drowsiness or weight gain (Cohen et al., 2020). Herbal decoction and acupuncture therapies exhibit satisfactory clinical efficacy. Current animal experiments have confirmed the relationship of acupuncture and inhibition of central inflammatory responses, regulation of neuronal autophagy activity levels, and promotion of benign expression of related proteins. Brain imaging-based studies have also indicated that acupuncture can improve patients' hemodynamic responses, increase connectivity between cognition-related regions and regulate brain networks, thereby improving their cognitive function (Tan et al., 2017; Khan et al., 2022). However, the prescription of these therapies needs to be guided by the core principle of TCM, syndrome differentiation, which undoubtedly increases the difficulty of their wide application.

The MMSE scale is a widely used test to screen for cognitive impairment; however, it has been found to be less sensitive than the MoCA scale in detecting VCIND, as it is less capable of testing for complex cognitive impairments in domains such as visuospatial and executive function (Dong et al., 2010). By comparison, the MoCA scale has been designed to detect mild cognitive abnormalities as it can detect complex cognitive impairments such as executive function and visual perception/construction, and its Attention and Delayed Recall test items are more challenging (Dong et al., 2010). Although the MMSE scale is inferior to the MOCA scale in screening for VCIND (Dong et al., 2010), it can compensate for the lack of specificity and positive predictive value of the MoCA scale during the screening process (Huang et al., 2021). Therefore, in this study, we used the MOCA scale as the primary outcome and the MMSE scale as the secondary outcome.

A previous meta-analysis by Cao and colleagues compared acupuncture with no treatment, placebo or conventional therapies (Cao et al., 2013). They speculated that acupuncture could be effective as an adjunctive treatment with cognitive function training or pharmaceutical therapies, but no firm conclusion for acupuncture can be drawn due to insufficient quality of evidence. An Updated meta-analysis by Deng and colleague evaluated acupuncture therapy, conventional therapy and pharmacotherapy in the treatment of VCIND, indicating acupuncture therapy to be a better choice (Deng and Wang, 2016). Their results cautiously suggested that acupuncture therapy can improve the clinical efficacy for VCIND.

Overall, our results have implications for VCIND, and there are a few caveats that should be noted. First, while the combination of manual acupuncture with herbal decoction demonstrated great potential for counteracting VCIND, it should still be noted that the application of this therapeutic regimen should follow the core principle of TCM, syndrome differentiation, as much as possible. Second, the absence of a uniform acupoint selection protocol is a major problem for the popularization of acupuncture therapies. Further efforts are warranted to determine the optimal acupoint selection scheme for VCIND. Finally, high quality RCTs are still needed to add robust evidence to further determine the optimal therapeutic strategies for patients with VCIND.

## Limitations

The findings of our meta-analysis must be considered in light of several limitations. First, a network meta-analysis inevitably shares the limitations of the trials included. To minimize the effects of this shortcoming, we only focused on randomized controlled trials. Second, the length of study period varied between trials. To overcome this limitation and minimize the impact of differences between observation periods, we recorded the outcomes as close to 3 months as possible for all analyses. If information at 3 months was not available, we used data ranging between 1 and 5 months (priority was given to the time point closest to 3 months; if equidistant, the longer one was chosen). Finally, our study did not take into account all available drugs for the treatment of VCIND, such as oxiracetam, Xingnaojing, etc., because they could not form a closed-loop relationship with other interventions in this network meta-analysis. More head-to-head RCTs comparing different drugs with acupuncture treatments for VCIND are required in the future.

The findings from this network meta-analysis represent the relatively comprehensive evidence base to guide the selection of treatment for VCIND. All statements should be tempered by consideration of the above limitations. We hope that these results will contribute to joint decision making between patients and their clinicians.

## Conclusion

In this network meta-analysis, manual acupuncture plus herbal decoction was shown to be the most effective intervention for VCIND in terms of the primary outcome MoCA. Manual acupuncture plus herbal decoction, electroacupuncture and manual acupuncture plus piracetam might be relatively ideal options for VCIND, whereas monotherapy with nimodipine or donepezil hydrochloride was considered to be the least efficacious intervention. Furthermore, the combination of acupuncture therapies and pharmacotherapy had a tendency to perform better than monotherapy in terms of clinical outcomes.

## Data availability statement

The original contributions presented in the study are included in the article/Supplementary material, further inquiries can be directed to the corresponding author.

## Author contributions

RL and PZ conceived and designed the study. RL, CX, and YL searched literature and selected the articles. RL, KW, and YL extracted the data. RL and PZ analyzed and interpreted the data. RL and CX wrote the first draft of the manuscript. XZ, JH, PZ, LX, and XD directed the manuscript and contributed to the writing of the final version of the manuscript. All authors agreed with the results and conclusions of this article.
