# Supplementary material for: Efficacy of acupuncture and pharmacological therapies for vascular cognitive impairment with no dementia: a network meta-analysis
Source: Front Aging Neurosci. 2023 Jun 15;15:1181160. doi: 10.3389/fnagi.2023.1181160 (PMC10310406; doi:10.3389/fnagi.2023.1181160)
Supplement: Supplementary file 1 [file Data_Sheet_1.docx]

**Supplementary Material**

Table S1. The Search strategy for PubMed.

| No | Search history | Results | Date |
| --- | --- | --- | --- |
| #1 | "Acupuncture"[Mesh] | 1,899 | 12 Mar 2022 |
| #2 | ((((pharmacopuncture[Title/Abstract]) OR (needle[Title/Abstract])) OR (needling[Title/Abstract])) OR (electroacupuncture[Title/Abstract])) OR (EA[Title/Abstract]) | 144,798 | 12 Mar 2022 |
| #3 | #1 OR #2 | 146,343 | 12 Mar 2022 |
| #4 | "Acupuncture Therapy"[Mesh] | 27,169 | 12 Mar 2022 |
| #5 | (((((((acupuncture treatment[Title/Abstract]) OR (acupuncture treatments[Title/Abstract])) OR (treatment, acupuncture[Title/Abstract])) OR (therapy, acupuncture[Title/Abstract])) OR (pharmacoacupuncture treatment[Title/Abstract])) OR (treatment, pharmacoacupuncture[Title/Abstract])) OR (pharmacoacupuncture therapy[Title/Abstract])) OR (therapy, pharmacoacupuncture[Title/Abstract]) | 3,516 | 12 Mar 2022 |
| #6 | #4 OR #5 | 27,954 | 12 Mar 2022 |
| #7 | "Acupuncture Points"[Mesh] | 7,487 | 12 Mar 2022 |
| #8 | ((((acupuncture point[Title/Abstract]) OR (point, acupuncture[Title/Abstract])) OR (points, acupuncture[Title/Abstract])) OR (acupoints[Title/Abstract])) OR (acupoint[Title/Abstract]) | 6,868 | 12 Mar 2022 |
| #9 | #7 OR #8 | 10,581 | 12 Mar 2022 |
| #10 | #3 OR #6 OR #9 | 165,973 | 12 Mar 2022 |
| #11 | "Cognitive Dysfunction"[Mesh] | 28,824 | 12 Mar 2022 |
| #12 | ((((((((((cognitive dysfunctions[Title/Abstract]) OR (dysfunction, cognitive[Title/Abstract])) OR (dysfunctions, cognitive[Title/Abstract])) OR (cognitive impairments[Title/Abstract])) OR (cognitive impairment[Title/Abstract])) OR (impairment, cognitive[Title/Abstract])) OR (impairments, cognitive[Title/Abstract])) OR (cognitive decline[Title/Abstract])) OR (cognitive declines[Title/Abstract])) OR (decline, cognitive[Title/Abstract])) OR (declines, cognitive[Title/Abstract]) | 111,505 | 12 Mar 2022 |
| #13 | #11 OR #12 | 117,882 | 12 Mar 2022 |
| #14 | mild[Title/Abstract] | 395,126 | 12 Mar 2022 |
| #15 | (no dementia[Title/Abstract]) OR (without dementia[Title/Abstract]) | 828 | 12 Mar 2022 |
| #16 | #14 OR #15 | 395,749 | 12 Mar 2022 |
| #17 | #13 AND #16 | 27,504 | 12 Mar 2022 |
| #18 | ((((((vascular cognitive impairment with no dementia[Title/Abstract]) OR (vascular cognitive impairment no dementia[Title/Abstract])) OR (vascular cognitive impairment without dementia[Title/Abstract])) OR (VCIND[Title/Abstract])) OR (vascular mild cognitive impairment[Title/Abstract])) OR (VMCI[Title/Abstract])) OR (poststroke cognitive impairment no dementia[Title/Abstract]) | 267 | 12 Mar 2022 |
| #19 | #17 OR #18 | 27,521 | 12 Mar 2022 |
| #20 | ((randomized controlled trial[Publication Type]) OR (randomized[Title/Abstract])) OR (placebo[Title/Abstract]) | 939,350 | 12 Mar 2022 |
| #21 | #10 AND #19 AND #20 | 44 | 12 Mar 2022 |

Table S2. The Search strategy for Embase.

| No | Search history | Results | Date |
| --- | --- | --- | --- |
| #1 | 'acupuncture'/exp | 51,889 | 12 Mar 2022 |
| #2 | 'pharmacopuncture':ti,ab | 317 | 12 Mar 2022 |
| #3 | 'needle':ti,ab | 163,843 | 12 Mar 2022 |
| #4 | 'needling':ti,ab | 4,508 | 12 Mar 2022 |
| #5 | 'electroacupuncture':ti,ab | 7,288 | 12 Mar 2022 |
| #6 | 'ea':ti,ab | 28,440 | 12 Mar 2022 |
| #7 | #1 OR #2 OR #3 OR #4 OR #5 OR #6 | 238,829 | 12 Mar 2022 |
| #8 | 'acupuncture therapy':ti,ab | 1,785 | 12 Mar 2022 |
| #9 | 'acupuncture treatment':ti,ab | 18,248 | 12 Mar 2022 |
| #10 | 'acupuncture treatments':ti,ab | 4,510 | 12 Mar 2022 |
| #11 | 'treatment, acupuncture':ti,ab | 205 | 12 Mar 2022 |
| #12 | 'therapy, acupuncture':ti,ab | 282 | 12 Mar 2022 |
| #13 | 'pharmacoacupuncture treatment':ti,ab | 1 | 12 Mar 2022 |
| #14 | 'treatment, pharmacoacupuncture':ti,ab | 0 | 12 Mar 2022 |
| #15 | 'pharmacoacupuncture therapy':ti,ab | 2 | 12 Mar 2022 |
| #16 | 'therapy, pharmacoacupuncture':ti,ab | 0 | 12 Mar 2022 |
| #17 | #8 OR #9 OR #10 OR #11 OR #12 OR #13 OR #14 OR #15 OR #16 | 19,774 | 12 Mar 2022 |
| #18 | 'acupuncture point'/exp | 3,198 | 12 Mar 2022 |
| #19 | 'acupuncture points':ti,ab | 2,128 | 12 Mar 2022 |
| #20 | 'point, acupuncture':ti,ab | 116 | 12 Mar 2022 |
| #21 | 'points, acupuncture':ti,ab | 41 | 12 Mar 2022 |
| #22 | 'acupoints':ti,ab | 4,632 | 12 Mar 2022 |
| #23 | 'acupoint':ti,ab | 4,082 | 12 Mar 2022 |
| #24 | #18 OR #19 OR #20 OR #21 OR #22 OR #23 | 10,511 | 12 Mar 2022 |
| #25 | #7 OR #17 OR #24 | 241,143 | 12 Mar 2022 |
| #26 | 'cognitive defect'/exp | 556,416 | 12 Mar 2022 |
| #27 | 'cognitive dysfunction':ti,ab | 24,006 | 12 Mar 2022 |
| #28 | 'cognitive dysfunctions':ti,ab | 2,484 | 12 Mar 2022 |
| #29 | 'dysfunction, cognitive':ti,ab | 335 | 12 Mar 2022 |
| #30 | 'dysfunctions, cognitive':ti,ab | 23 | 12 Mar 2022 |
| #31 | 'cognitive impairments':ti,ab | 14,945 | 12 Mar 2022 |
| #32 | 'cognitive impairment':ti,ab | 106,604 | 12 Mar 2022 |
| #33 | 'impairment, cognitive':ti,ab | 570 | 12 Mar 2022 |
| #34 | 'impairments, cognitive':ti,ab | 112 | 12 Mar 2022 |
| #35 | 'cognitive decline':ti,ab | 41,590 | 12 Mar 2022 |
| #36 | 'cognitive declines':ti,ab | 551 | 12 Mar 2022 |
| #37 | 'decline, cognitive':ti,ab | 130 | 12 Mar 2022 |
| #38 | 'declines, cognitive':ti,ab | 3 | 12 Mar 2022 |
| #39 | #26 OR #27 OR #28 OR #29 OR #30 OR #31 OR #32 OR #33 OR #34 OR #35 OR #36 OR #37 OR #38 | 584,750 | 12 Mar 2022 |
| #40 | 'mild':ti,ab | 601,295 | 12 Mar 2022 |
| #41 | 'no dementia':ti,ab | 1,195 | 12 Mar 2022 |
| #42 | 'without dementia':ti,ab | 5,571 | 12 Mar 2022 |
| #43 | #40 OR #41 OR #42 | 606,745 | 12 Mar 2022 |
| #44 | #39 AND #43 | 63,134 | 12 Mar 2022 |
| #45 | 'vascular cognitive impairment with no dementia':ti,ab | 23 | 12 Mar 2022 |
| #46 | 'vascular cognitive impairment no dementia':ti,ab | 115 | 12 Mar 2022 |
| #47 | 'vascular cognitive impairment without dementia':ti,ab | 18 | 12 Mar 2022 |
| #48 | 'vcind':ti,ab | 156 | 12 Mar 2022 |
| #49 | 'vascular mild cognitive impairment':ti,ab | 181 | 12 Mar 2022 |
| #50 | 'vmci':ti,ab | 72 | 12 Mar 2022 |
| #51 | 'poststroke cognitive impairment no dementia':ti,ab | 5 | 12 Mar 2022 |
| #52 | #45 OR #46 OR #47 OR #48 OR #49 OR #50 OR #51 | 413 | 12 Mar 2022 |
| #53 | #44 OR #52 | 63,158 | 12 Mar 2022 |
| #54 | 'random':ti,ab | 379,855 | 12 Mar 2022 |
| #55 | 'placebo':ti,ab | 338,385 | 12 Mar 2022 |
| #56 | 'double-blind':ti,ab | 211,272 | 12 Mar 2022 |
| #57 | #54 OR #55 OR #56 | 774,696 | 12 Mar 2022 |
| #58 | #25 AND #53 AND #57 | 21 | 12 Mar 2022 |

Table S3. The Search strategy for WOS.

| No | Search history | Results | Date |
| --- | --- | --- | --- |
| #1 | TS=(acupuncture OR pharmacopuncture OR needle OR needling OR electroacupuncture OR EA OR acupuncture therapy OR acupuncture treatment OR acupuncture treatments OR treatment, acupuncture OR therapy, acupuncture OR pharmacoacupuncture treatment OR treatment, pharmacoacupuncture OR pharmacoacupuncture therapy OR therapy, pharmacoacupuncture OR acupuncture points OR acupuncture point OR point, acupuncture OR points, acupuncture OR acupoints OR acupoint) | 826,395 | 12 Mar 2022 |
| #2 | TS=(((cognitive dysfunction OR cognitive dysfunctions OR dysfunction, cognitive OR dysfunctions, cognitive OR cognitive impairments OR cognitive impairment OR impairment, cognitive OR impairments, cognitive OR cognitive decline OR cognitive declines OR decline, cognitive OR declines, cognitive) AND (mild OR no dementia OR without dementia)) OR (vascular cognitive impairment with no dementia OR vascular cognitive impairment no dementia OR vascular cognitive impairment without dementia OR VCIND OR vascular mild cognitive impairment OR VMCI OR poststroke cognitive impairment no dementia)) | 74,992 | 12 Mar 2022 |
| #3 | TS=(random* controlled trial OR random* OR placebo) | 3,248,985 | 12 Mar 2022 |
| #4 | #1 AND #2 AND #3 | 126 | 12 Mar 2022 |

Table S4. The Search strategy for the Cochrane Library.

| No | Search history | Results | Date |
| --- | --- | --- | --- |
| #1 | MeSH descriptor: [Acupuncture] explode all trees | 158 | 12 Mar 2022 |
| #2 | (pharmacopuncture):ti,ab,kw OR (needle):ti,ab,kw OR (needling):ti,ab,kw OR (electroacupuncture):ti,ab,kw OR (EA):ti,ab,kw | 21,632 | 12 Mar 2022 |
| #3 | #1 or #2 | 21,749 | 12 Mar 2022 |
| #4 | MeSH descriptor: [Acupuncture Therapy] explode all trees | 5,173 | 12 Mar 2022 |
| #5 | (acupuncture treatment):ti,ab,kw OR (acupuncture treatments):ti,ab,kw OR (treatment, acupuncture):ti,ab,kw OR (therapy, acupuncture):ti,ab,kw OR (pharmacoacupuncture treatment):ti,ab,kw OR (treatment, pharmacoacupuncture):ti,ab,kw OR (pharmacoacupuncture therapy):ti,ab,kw OR (therapy, pharmacoacupuncture):ti,ab,kw | 12,745 | 12 Mar 2022 |
| #6 | #4 or #5 | 13,236 | 12 Mar 2022 |
| #7 | MeSH descriptor: [Acupuncture Points] explode all trees | 2,212 | 12 Mar 2022 |
| #8 | (acupuncture point):ti,ab,kw OR (point, acupuncture):ti,ab,kw OR (points, acupuncture):ti,ab,kw OR (acupoints):ti,ab,kw OR (acupoint):ti,ab,kw | 8,487 | 12 Mar 2022 |
| #9 | #7 or #8 | 8,487 | 12 Mar 2022 |
| #10 | #3 or #6 or #9 | 32,626 | 12 Mar 2022 |
| #11 | MeSH descriptor: [Cognitive Dysfunction] explode all trees | 2,115 | 12 Mar 2022 |
| #12 | (cognitive dysfunctions):ti,ab,kw OR (dysfunction, cognitive):ti,ab,kw OR (dysfunctions, cognitive):ti,ab,kw OR (cognitive impairments):ti,ab,kw OR (cognitive impairment):ti,ab,kw OR (impairment, cognitive):ti,ab,kw OR (impairments, cognitive):ti,ab,kw OR (cognitive decline):ti,ab,kw OR (cognitive declines):ti,ab,kw OR (decline, cognitive):ti,ab,kw OR (declines, cognitive):ti,ab,kw | 22,225 | 12 Mar 2022 |
| #13 | #11 or #12 | 22,234 | 12 Mar 2022 |
| #14 | (mild):ti,ab,kw OR (no dementia):ti,ab,kw OR (without dementia):ti,ab,kw | 72,750 | 12 Mar 2022 |
| #15 | #13 and #14 | 6,409 | 12 Mar 2022 |
| #16 | (vascular cognitive impairment with no dementia):ti,ab,kw OR (vascular cognitive impairment no dementia):ti,ab,kw OR (vascular cognitive impairment without dementia):ti,ab,kw OR (VCIND):ti,ab,kw OR (vascular mild cognitive impairment):ti,ab,kw OR (VMCI):ti,ab,kw OR (poststroke cognitive impairment no dementia):ti,ab,kw | 448 | 12 Mar 2022 |
| #1 | MeSH descriptor: [Acupuncture] explode all trees | 158 | 12 Mar 2022 |
| #2 | (pharmacopuncture):ti,ab,kw OR (needle):ti,ab,kw OR (needling):ti,ab,kw OR (electroacupuncture):ti,ab,kw OR (EA):ti,ab,kw | 21,632 | 12 Mar 2022 |
| #3 | #1 or #2 | 21,749 | 12 Mar 2022 |
| #4 | MeSH descriptor: [Acupuncture Therapy] explode all trees | 5,173 | 12 Mar 2022 |
| #5 | (acupuncture treatment):ti,ab,kw OR (acupuncture treatments):ti,ab,kw OR (treatment, acupuncture):ti,ab,kw OR (therapy, acupuncture):ti,ab,kw OR (pharmacoacupuncture treatment):ti,ab,kw OR (treatment, pharmacoacupuncture):ti,ab,kw OR (pharmacoacupuncture therapy):ti,ab,kw OR (therapy, pharmacoacupuncture):ti,ab,kw | 12,745 | 12 Mar 2022 |
| #6 | #4 or #5 | 13,236 | 12 Mar 2022 |
| #7 | MeSH descriptor: [Acupuncture Points] explode all trees | 2,212 | 12 Mar 2022 |
| #8 | (acupuncture point):ti,ab,kw OR (point, acupuncture):ti,ab,kw OR (points, acupuncture):ti,ab,kw OR (acupoints):ti,ab,kw OR (acupoint):ti,ab,kw | 8,487 | 12 Mar 2022 |
| #9 | #7 or #8 | 8,487 | 12 Mar 2022 |
| #10 | #3 or #6 or #9 | 32,626 | 12 Mar 2022 |
| #11 | MeSH descriptor: [Cognitive Dysfunction] explode all trees | 2,115 | 12 Mar 2022 |
| #12 | (cognitive dysfunctions):ti,ab,kw OR (dysfunction, cognitive):ti,ab,kw OR (dysfunctions, cognitive):ti,ab,kw OR (cognitive impairments):ti,ab,kw OR (cognitive impairment):ti,ab,kw OR (impairment, cognitive):ti,ab,kw OR (impairments, cognitive):ti,ab,kw OR (cognitive decline):ti,ab,kw OR (cognitive declines):ti,ab,kw OR (decline, cognitive):ti,ab,kw OR (declines, cognitive):ti,ab,kw | 22,225 | 12 Mar 2022 |
| #13 | #11 or #12 | 22,234 | 12 Mar 2022 |
| #14 | (mild):ti,ab,kw OR (no dementia):ti,ab,kw OR (without dementia):ti,ab,kw | 72,750 | 12 Mar 2022 |
| #15 | #13 and #14 | 6,409 | 12 Mar 2022 |
| #16 | (vascular cognitive impairment with no dementia):ti,ab,kw OR (vascular cognitive impairment no dementia):ti,ab,kw OR (vascular cognitive impairment without dementia):ti,ab,kw OR (VCIND):ti,ab,kw OR (vascular mild cognitive impairment):ti,ab,kw OR (VMCI):ti,ab,kw OR (poststroke cognitive impairment no dementia):ti,ab,kw | 448 | 12 Mar 2022 |
| #17 | #15 or #16 | 6,415 | 12 Mar 2022 |
| #18 | #10 and #17 | 117 | 12 Mar 2022 |

Table S5. Characteristics of included patients.

| Study ID | Main Country | Number of patients  (E/C) | Sex  (M/F) | Mean age  (years)(E/C) | Course of disease  (d,days; m,months; y,years) |
| --- | --- | --- | --- | --- | --- |
| Yu 2007 | China | 31/33 | 33/31 | 67.24±2.17/  66.98±2.32 | 0.83±0.13y/  0.88±0.21y |
| Jiao 2011 | China | 30/30 | 34/26 | 62.2/  63.4 | 10.8±15.1m/  10.0±13.0m |
| Kong 2011 | China | 30/30 | 39/21 | 58.17±7.23/  57.13±6.35 | 7.03±2.47m/  6.83±2.51m |
| Li 2012 | China | 48/46 | 54/40 | 68.29±8.22/  69.22±7.88 | 13.33±9.58m/  12.26±7.82m |
| Shao 2012 | China | 30/30 | 35/25 | 63.37±3.886/  66.13±8.885 | 12.57±1.165m/  12.53±1.252m |
| Feng 2013 | China | 24/24 | 28/20 | 62.54±7.22/  63.21±7.16 | 5.35±2.61m/  5.12±2.75m |
| Chen 2014 | China | 20/20 | 20/20 | 67.05±10.70/  69.45±9.29 | - |
| Wang 2014 | China | 30/30 | 31/29 | - | - |
| Zhang 2014 | China | 30/30 | 33/27 | 70.90±5.62/  71.27±5.91 | - |
| Zheng 2014 | China | 30/30 | 36/24 | 68.33±8.33/  66.83±8.55 | 3.10±1.45m/  3.33±1.42m |
| Dong 2015 | China | 40/40 | 45/35 | - | - |
| Yang 2015 | China | 36/36 | 46/26 | 66±3/  66±3 | - |
| Sun 2016 | China | 40/40 | 41/39 | - | - |
| Wang 2016 | China | 31/30 | 29/32 | 63.03±5.67/  65.40±5.46 | 6.84±3.39m/  6.53±3.18m |
| Wang 2016 | China | 40/40/39 | 82/37 | 64.4±7.7/  65.2±7.1/  60.6±6.7 | - |
| Liu 2017 | China | 60/30 | 53/37 | 64±7/64±7 | 6.58±1.41m/  5.78±1.68m |
| Sun 2017 | China | 30/30 | 38/22 | 68.20±7.01/  70.40±6.58 | 4.617±0.827m/  4.567±0.666m |
| Wang 2017 | China | 20/20 | 19/21 | 64.2±7.05/  63.5±6.69 | - |
| Yu 2017 | China | 27/28 | 34/21 | 62.75±8.51/  63.35±7.32 | - |
| Zhao 2017 | China | 17/17 | - | - | - |
| Li 2018 | China | 30/30 | 33/27 | 63.63±6.91/  65.63±5.64 | 6.34±2.32m/  6.22±2.25m |
| Zhang 2018 | China | 30/30 | 27/33 | 58.17±6.539/  59.03±6.066 | - |
| Zhang 2018 | China | 40/40 | 39/41 | 71.00±2.74/  71.47±2.83 | 12.40±4.40m/  11.90±4.29m |
| Wang 2018 | China | 64/64 | 62/66 | 71.42±8.67/  69.33±7.56 | 67.57±19.42d/  63.34±17.37d |
| Luo 2019 | China | 94/94 | 112/76 | 71±7/  70±7 | 66.81±31.89d/  67.71±33.10d |
| Xu 2019 | China | 56/38 | 49/45 | 57.73±4.69/  56.97±5.35 | 1.86±0.37y/  1.83±0.46y |
| Zhang 2019 | China | 42/41/39 | 55/67 | 58.96±8.23/  58.55±7.83/  59.76±7.33 | 2.63±0.54y/  2.59±0.72y/  2.51±0.61y |
| Meng 2020 | China | 61/61 | 68/54 | 65.2±3.7/  65.6±3.5 | - |
| Ni 2020 | China | 30/30/30 | 55/35 | 59±7/  60±7/  61±7 | 5.60±1.59m/  6.17±2.03m/  6.00±1.66m |
| Yu 2020 | China | 40/40 | 47/33 | 63.54±7.28/  64.09±6.84 | - |
| Bai 2021 | China | 31/30 | 32/29 | 65.39±6.50/  64.93±5.69 | 1.32±0.46m/  1.40±0.50m |
| Liu 2021 | China | 34/34/34 | 49/53 | 57.09±10.54/  58.15±9.94/  58.76±10.18 | - |
| Zhang 2021 | China | 34/34 | 35/33 | 65.7±4.7/  67.2±5.1 | 48.68±11.38d/  47.46±9.75d |
| E: experimental group; C: control group.  M: male; F: female. | | | | | |

Table S6. Heterogeneity test results for MoCA.

| Interventions | Pair−wise effect (pooled) | Network effect  (pooled) | I^2^  (pair−wise) | I^2^  (network) | P value |
| --- | --- | --- | --- | --- | --- |
| B vs A | 3.00  (1.40, 4.50) | 2.30  (1.10, 3.50) | NA | 54.03 | 0.18 |
| C vs A | -2.10  (-3.00, -1.20) | -1.80  (-2.70, -0.91) | 0.00 | 96.84 | 0.00 |
| D vs A | -1.90  (-3.10, -0.74) | -1.90  (-3.40, -0.57) | 25.17 | 30.57 | NA |
| E vs A | -0.52  (-1.60, 0.46) | -0.90  (-2.10, 0.21) | 77.48 | 83.47 | NA |
| F vs A | 1.20  (-0.70, 3.20) | 1.20  (-0.78, 3.30) | NA | NA | NA |
| H vs A | -0.93  (-3.00, -1.10) | 0.08  (-1.00, 1.20) | NA | 32.26 | 0.26 |
| J vs A | 2.20  (0.94, 3.40) | 1.70  (0.19, 3.10) | NA | NA | NA |
| K vs A | 4.10  (1.80, 6.40) | 4.10  (2.00, 6.20) | NA | NA | NA |
| C vs B | -3.10  (-4.30, -1.90) | -4.10  (-5.30, -2.90) | NA | NA | NA |
| E vs B | -4.50  (-6.00, -3.00) | -3.20  (-4.70, -1.80) | NA | 96.12 | 0.00 |
| G vs C | 2.00  (1.20, 2.80) | 2.00  (1.00, 3.00) | 0.00 | 0.00 | NA |
| H vs C | 1.80  (1.20, 2.60) | 1.80  (1.10, 2.60) | 70.70 | 70.89 | NA |
| L vs C | 1.60  (0.21, 2.90) | 1.60  (-0.14, 3.30) | NA | NA | NA |
| M vs C | 1.70  (0.45, 3.00) | 1.70  (0.08, 3.40) | NA | NA | NA |
| N vs C | 0.92  (-0.43, 2.30) | 0.94  (-0.77, 2.60) | NA | NA | NA |
| I vs D | 1.20  (0.68, 1.80) | 1.30  (0.55, 2.00) | 0.00 | 0.00 | NA |
| J vs E | 2.10  (0.85, 3.30) | 2.60  (1.10, 4.00) | NA | NA | NA |
| K vs F | 2.90  (0.88, 4.80) | 2.90  (0.84, 4.90) | NA | NA | NA |

Intervention: A: manual acupuncture; B: electroacupuncture; C: nimodipine; D: donepezil hydrochloride; E: piracetam; F: herbal decoction; G: manual acupuncture plus electroacupuncture; H: manual acupuncture plus nimodipine; I: manual acupuncture plus donepezil hydrochloride; J: manual acupuncture plus piracetam; K: manual acupuncture plus herbal decoction; L: electroacupuncture plus nimodipine; M: warm acupuncture plus nimodipine; N: manual acupuncture plus electroacupuncture plus nimodipine.

Table S7. The results of Node-splitting test, the assessment of consistency and GRADE quality of evidence for MoCA.

| Interventions | Direct comparison | | Indirect comparison | | Network comparison | | P value |
| --- | --- | --- | --- | --- | --- | --- | --- |
|  | WMD (95%CI) | Quality of evidence | WMD (95%CI) | Quality of evidence | WMD (95%CI) | Quality of evidence |  |
| A vs B | 3.00  (1.70, 4.30) | Moderate | 0.89  (-0.46, 2.20) | Moderate | 2.30  (1.10, 3.50) | Moderate | 0.03 |
| A vs C | -2.10  (-3.00, -1.20) | Moderate | 0.40  (-1.50, 2.30) | Low | -1.80  (-2.70, -0.92) | Moderate | 0.02 |
| A vs H | -0.89  (-3.10, 1.30) | Low | 0.43  (-0.88, 1.70) | Moderate | 0.084  (-1.00, 1.20) | Moderate | 0.29 |
| B vs C | -3.10  (-4.30, -1.80) | Moderate | -5.70  (-7.20, -4.10) | Moderate | -4.10  (-5.40, -2.90) | Moderate | 0.02 |
| B vs E | -4.50  (-5.80, -3.20) | Moderate | -0.80  (-2.50, 0.92) | Very low | -3.20  (-4.70, -1.80) | Moderate | 0.00 |

Intervention: A: manual acupuncture; B: electroacupuncture; C: nimodipine; E: piracetam; H: manual acupuncture plus nimodipine.


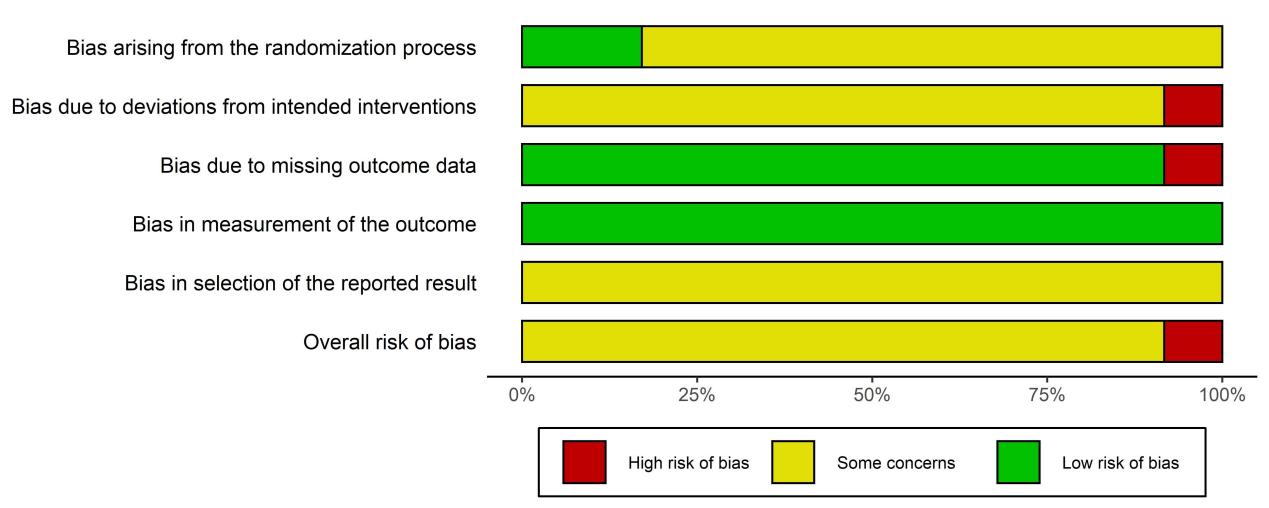


Figure S1. Risk of bias graph.


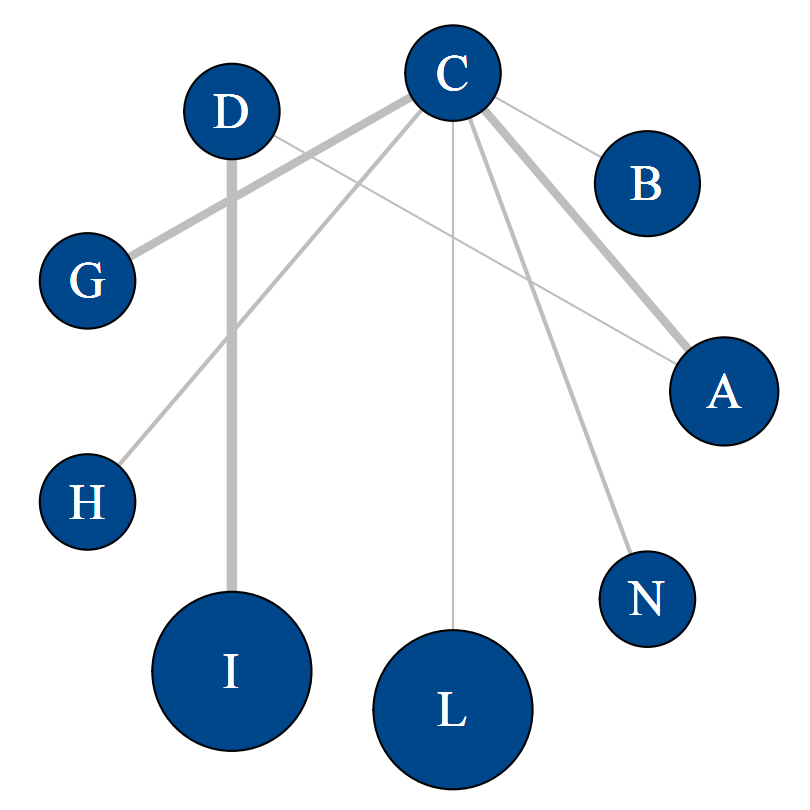


Figure S2. Network map of different interventions for MMSE.

Width of the lines is proportional to the number of trials comparing every pair of treatments. Size of every circle is proportional to the number of randomly assigned participants (i.e., sample size). Intervention: A: manual acupuncture; B: electroacupuncture; C: nimodipine; D: donepezil hydrochloride; G: manual acupuncture plus electroacupuncture; H: manual acupuncture plus nimodipine; I: manual acupuncture plus donepezil hydrochloride; L: electroacupuncture plus nimodipine; N: manual acupuncture plus electroacupuncture plus nimodipine.


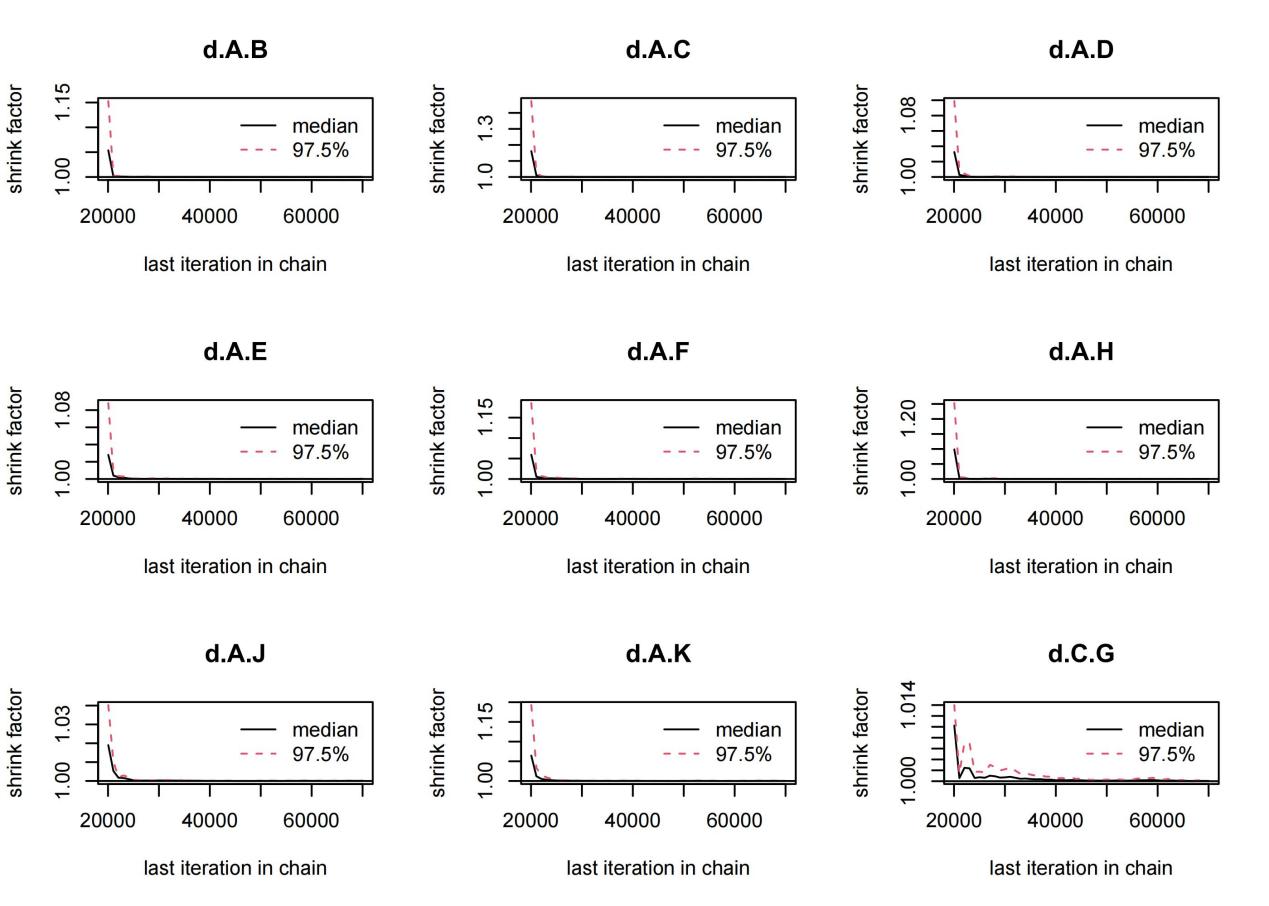


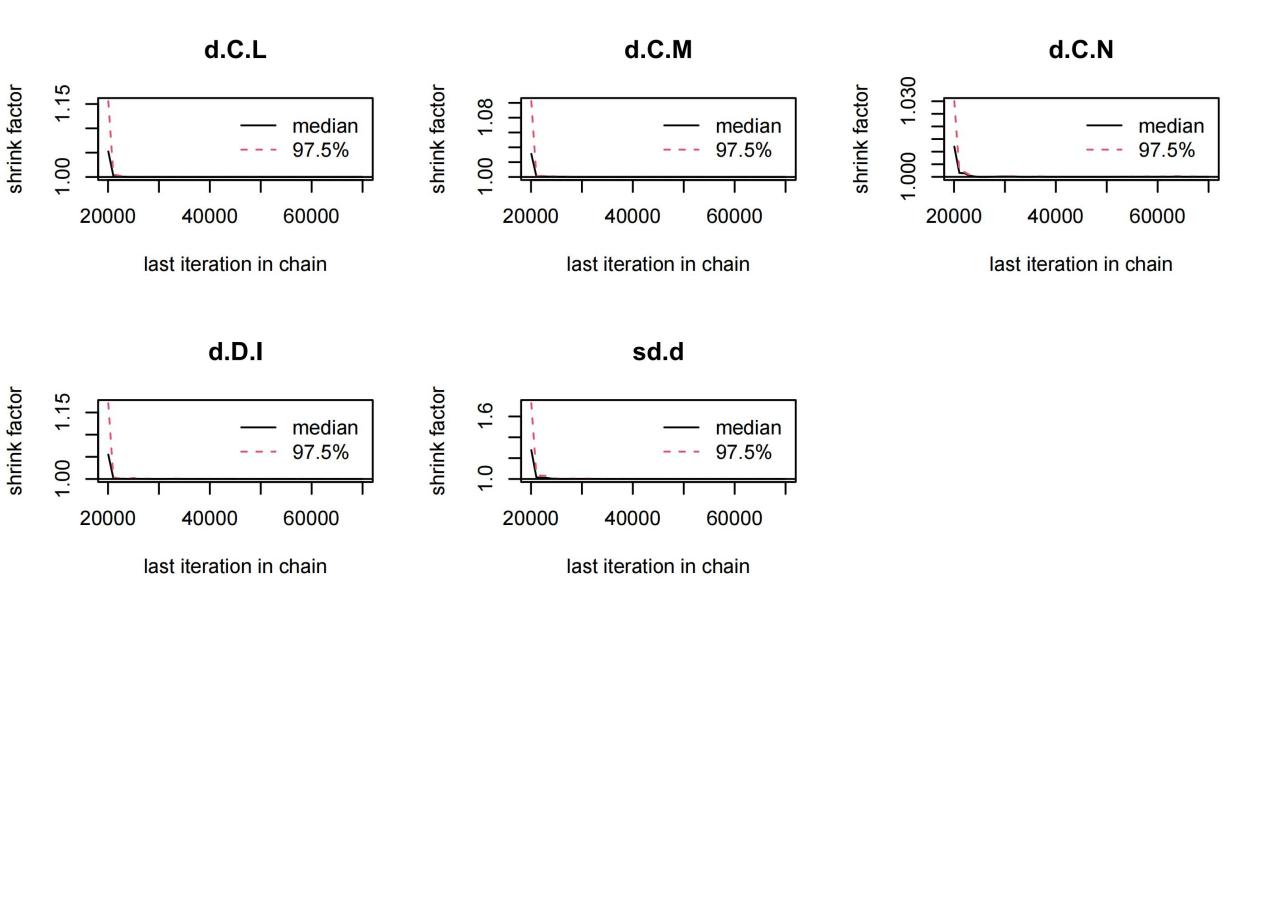


Figure S3. The convergence diagnostic plot of the model for MoCA.

Intervention: A: manual acupuncture; B: electroacupuncture; C: nimodipine; D: donepezil hydrochloride; E: piracetam; F: herbal decoction; G: manual acupuncture plus electroacupuncture; H: manual acupuncture plus nimodipine; I: manual acupuncture plus donepezil hydrochloride; J: manual acupuncture plus piracetam; K: manual acupuncture plus herbal decoction; L: electroacupuncture plus nimodipine; M: warm acupuncture plus nimodipine; N: manual acupuncture plus electroacupuncture plus nimodipine.


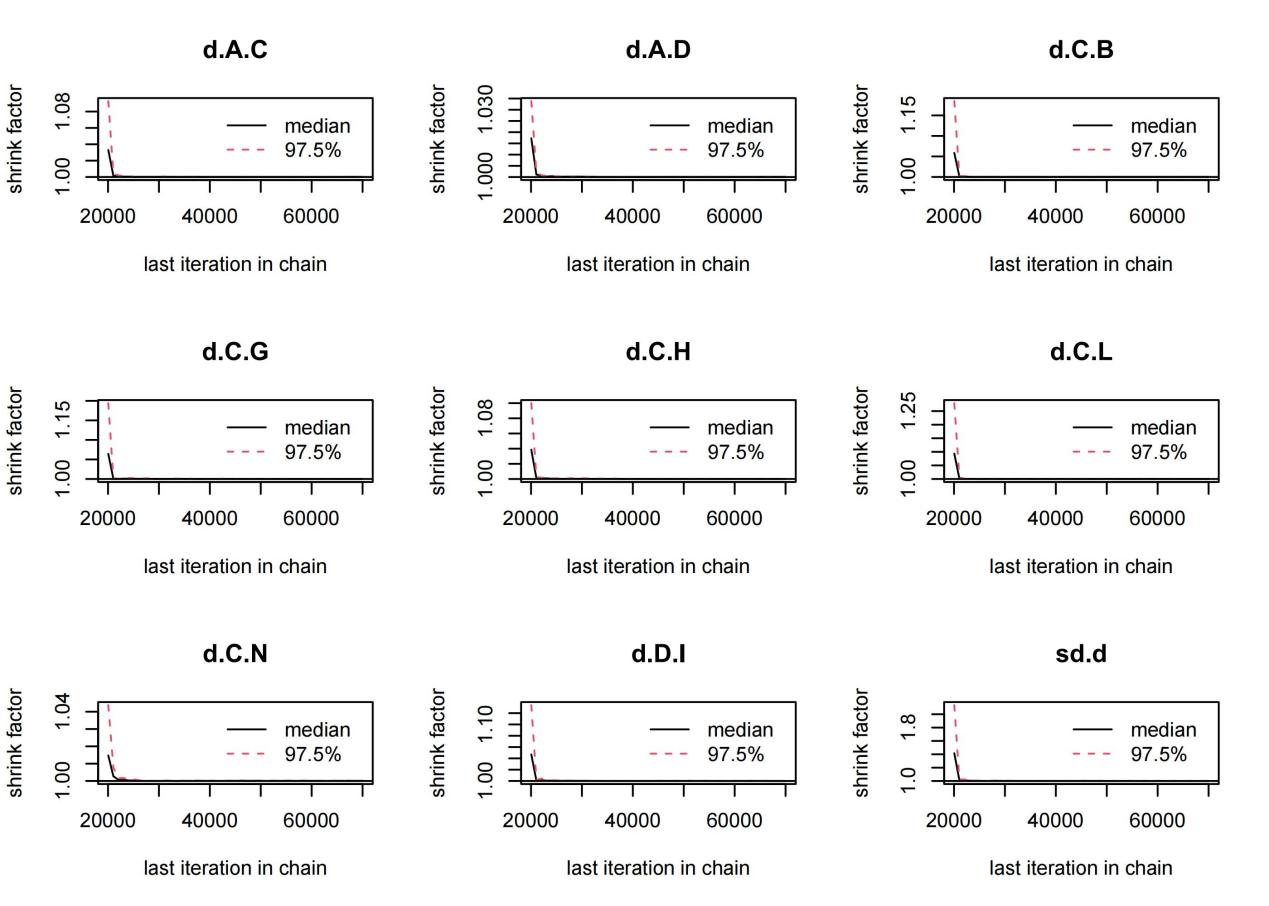


Figure S4. The convergence diagnostic plot of the model for MMSE.

Intervention: A: manual acupuncture; B: electroacupuncture; C: nimodipine; D: donepezil hydrochloride; G: manual acupuncture plus electroacupuncture; H: manual acupuncture plus nimodipine; I: manual acupuncture plus donepezil hydrochloride; L: electroacupuncture plus nimodipine; N: manual acupuncture plus electroacupuncture plus nimodipine.


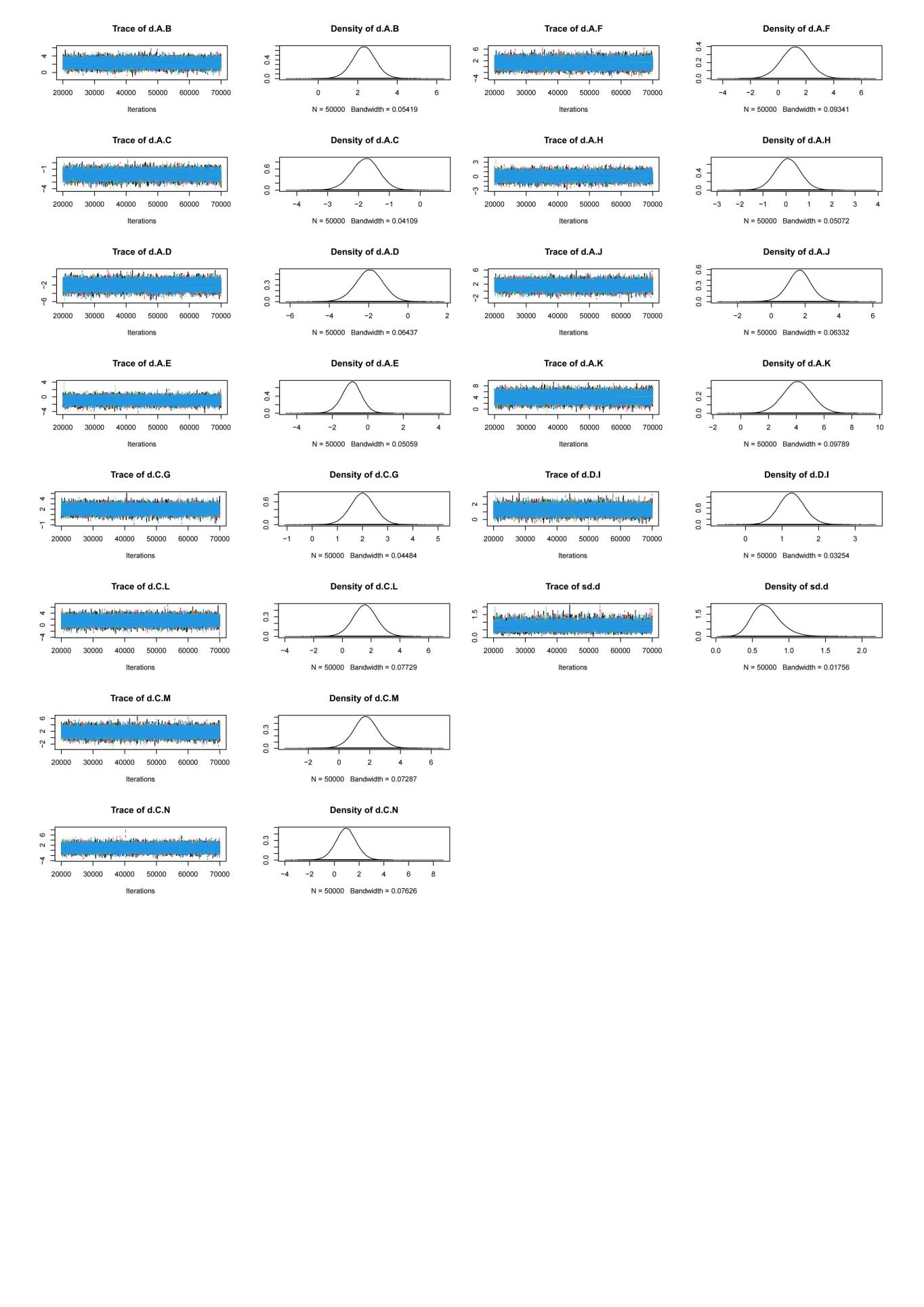


Figure S5. The density plot and trace plot of the model for MoCA.

Intervention: A: manual acupuncture; B: electroacupuncture; C: nimodipine; D: donepezil hydrochloride; E: piracetam; F: herbal decoction; G: manual acupuncture plus electroacupuncture; H: manual acupuncture plus nimodipine; I: manual acupuncture plus donepezil hydrochloride; J: manual acupuncture plus piracetam; K: manual acupuncture plus herbal decoction; L: electroacupuncture plus nimodipine; M: warm acupuncture plus nimodipine; N: manual acupuncture plus electroacupuncture plus nimodipine.


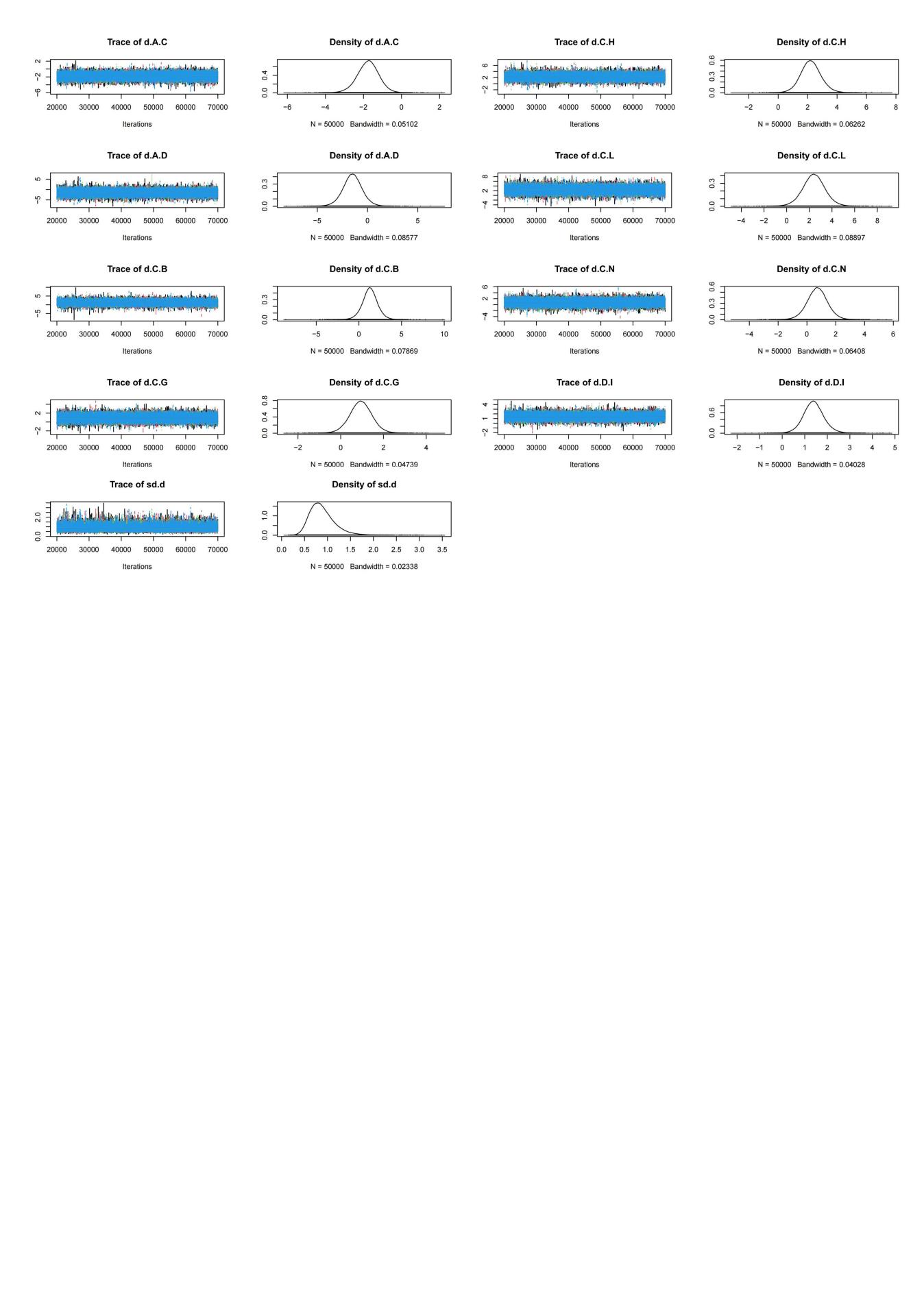


Figure S6. The density plot and trace plot of the model for MMSE.

Intervention: A: manual acupuncture; B: electroacupuncture; C: nimodipine; D: donepezil hydrochloride; G: manual acupuncture plus electroacupuncture; H: manual acupuncture plus nimodipine; I: manual acupuncture plus donepezil hydrochloride; L: electroacupuncture plus nimodipine; N: manual acupuncture plus electroacupuncture plus nimodipine.


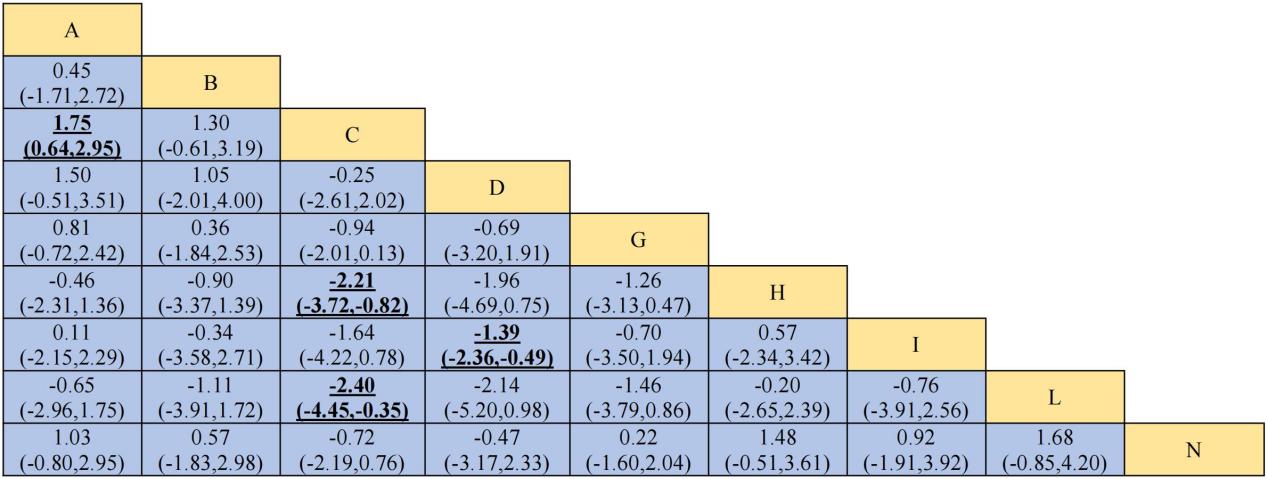


Figure S7. Network meta-analysis comparisons for MMSE.

Interventions are reported in alphabetical order. Data are MDs (95% CI) of the column-defining intervention compared to the row-defining intervention. For MMSE, 95% CI doesn’t contain 0 and MDs higher than 0 favor the column-defining treatment (i.e., the first in alphabetical order). Significant results are in bold and underscored. Nimodipine (MDs ranging between -1.75 and -2.40) and donepezil hydrochloride (MD is -1.39) are among the least efficacious interventions. MD: mean difference. CI: confidence interval. Intervention: A: manual acupuncture; B: electroacupuncture; C: nimodipine; D: donepezil hydrochloride; G: manual acupuncture plus electroacupuncture; H: manual acupuncture plus nimodipine; I: manual acupuncture plus donepezil hydrochloride; L: electroacupuncture plus nimodipine; N: manual acupuncture plus electroacupuncture plus nimodipine.


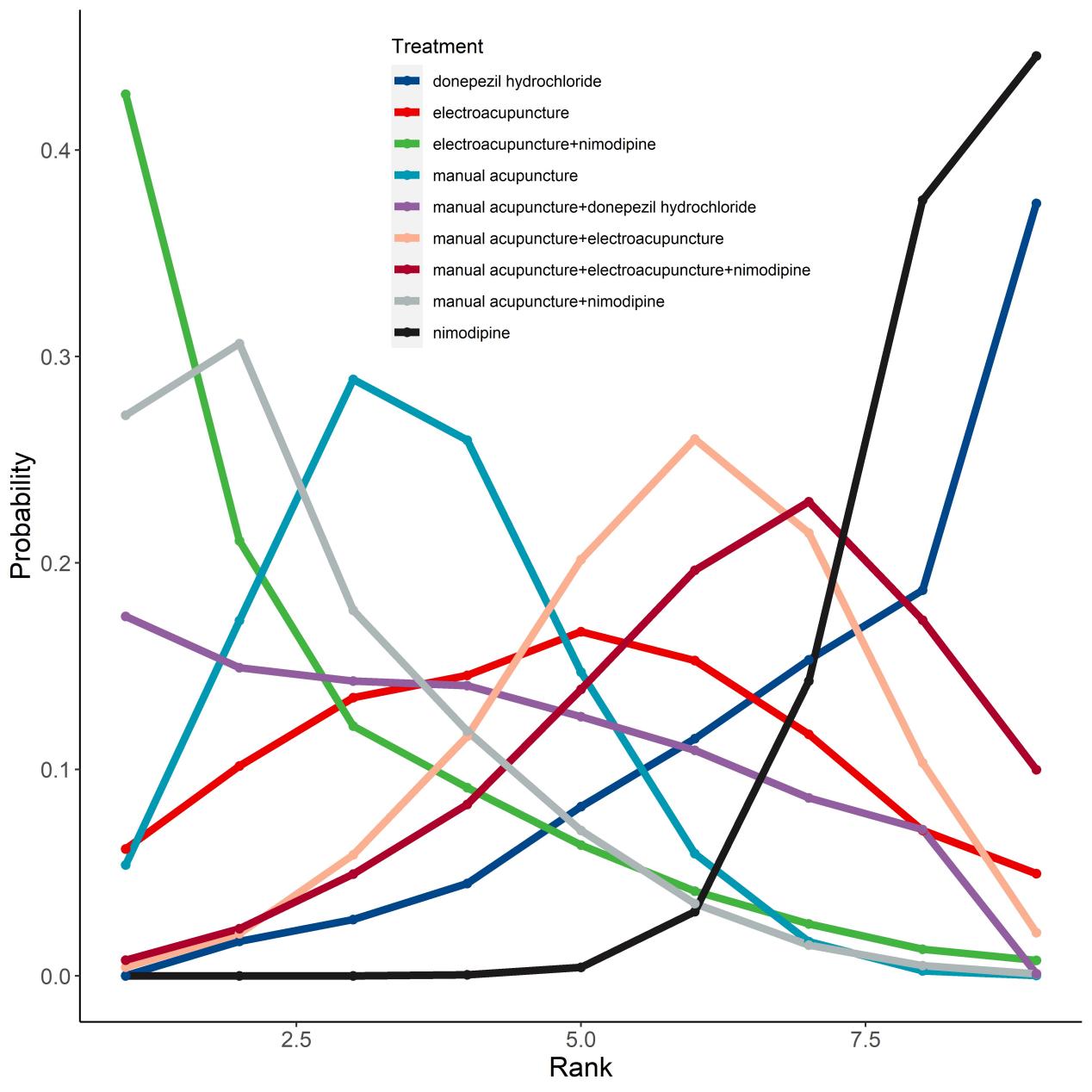


Figure S8. The rank probability of MMSE for included interventions.

Electroacupuncture plus nimodipine is the optimal strategy for improving MMSE scores (Probability of rank, 43%). Manual acupuncture plus nimodipine and manual acupuncture rank second to third, respectively. Nimodipine is the worst strategy in improving MMSE scores (Probability of rank, 45%).


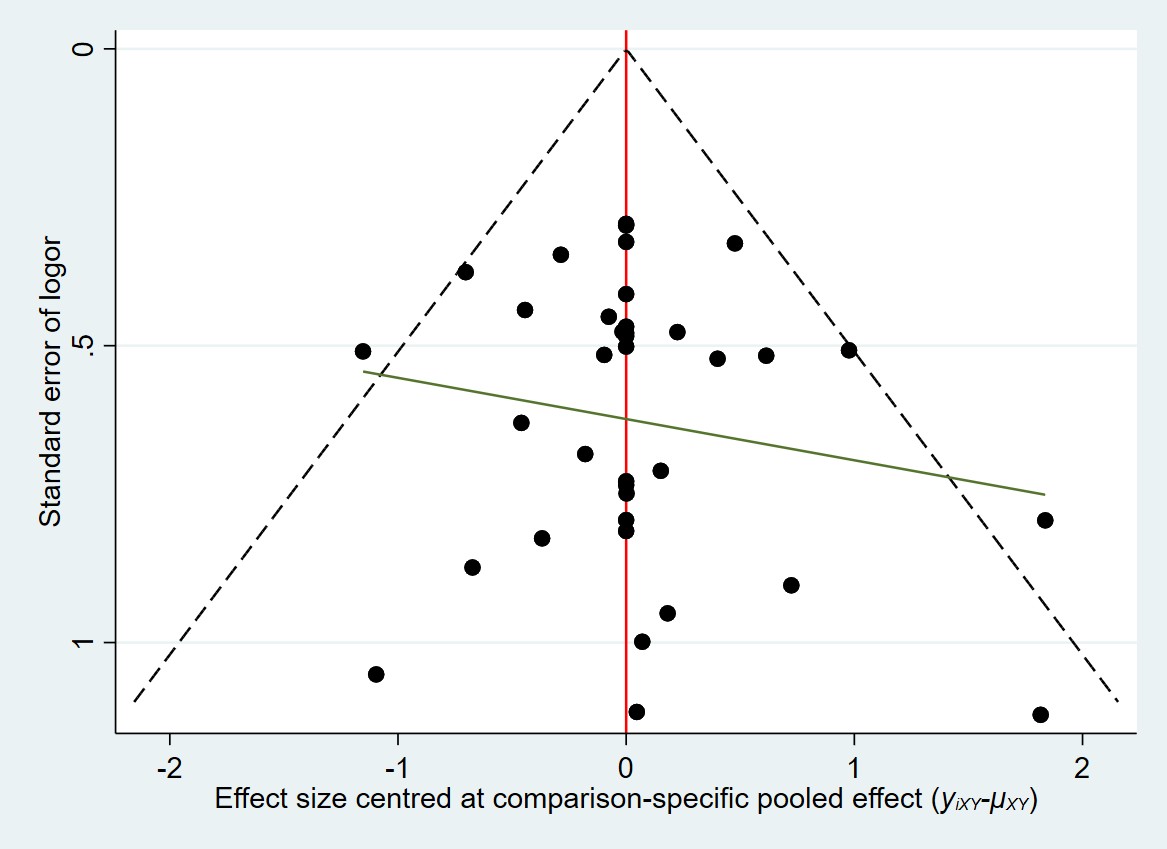


Figure S9. funnel plot of MoCA.


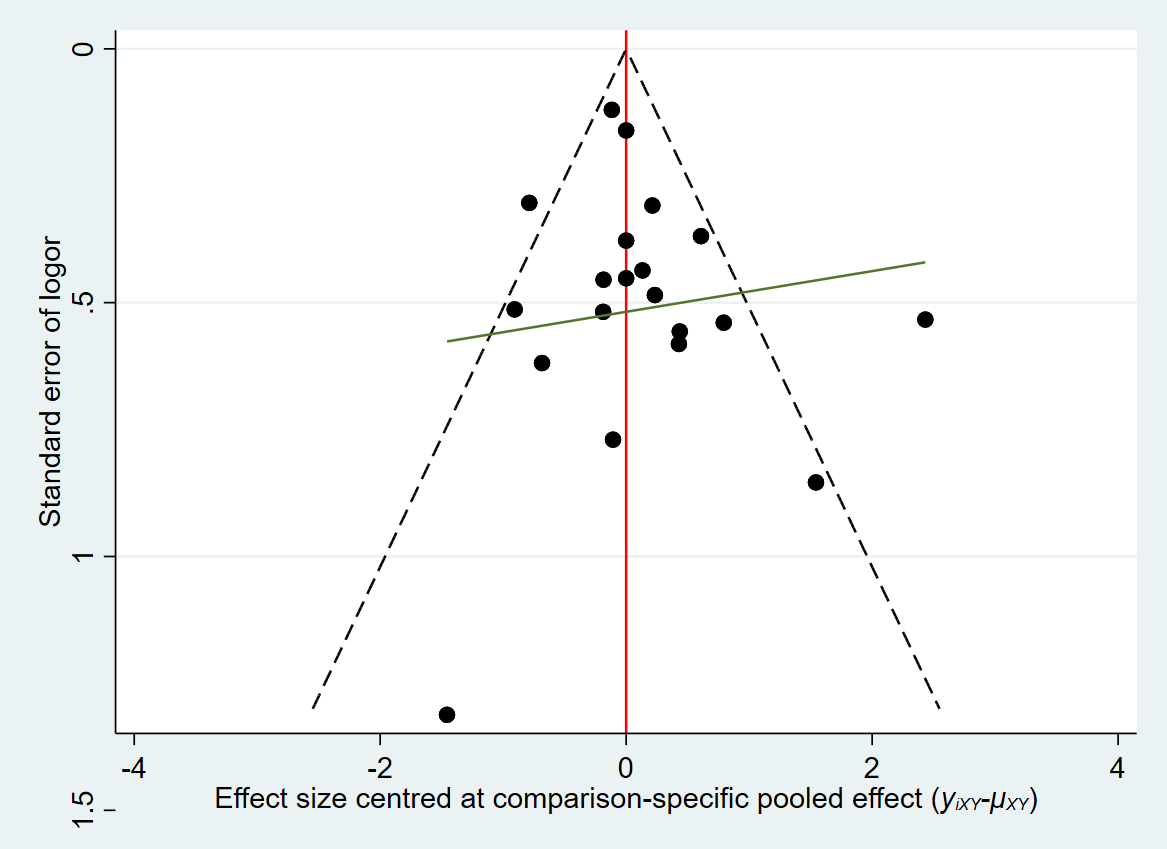


Figure S10. funnel plot of MMSE.

Table S1. The Search strategy for PubMed. docx

Table S2. The Search strategy for Embase. docx

Table S3. The Search strategy for WOS. docx

Table S4. The Search strategy for the Cochrane Library. docx

Table S5. Characteristics of included patients. docx

Table S6. Heterogeneity test results for MoCA. docx

Table S7. The results of Node-splitting test, the assessment of consistency and GRADE quality of evidence for MoCA. docx

Figure S1. Risk of bias graph. jpg

Figure S2. Network map of different interventions for MMSE. jpg

Figure S3. The convergence diagnostic plot of the model for MoCA. jpg

Figure S4. The convergence diagnostic plot of the model for MMSE. jpg

Figure S5. The density plot and trace plot of the model for MoCA. jpg

Figure S6. The density plot and trace plot of the model for MMSE. jpg

Figure S7. Network meta-analysis comparisons for MMSE. jpg

Figure S8. The rank probability of MMSE for included interventions. jpg

Figure S9. funnel plot of MoCA. jpg

Figure S10. funnel plot of MMSE. jpg
